# Causal Inference for Genetic Obesity, Cardiometabolic Profile and COVID-19 Susceptibility: A Mendelian Randomization Study

**DOI:** 10.3389/fgene.2020.586308

**Published:** 2020-11-11

**Authors:** Nay Aung, Mohammed Y. Khanji, Patricia B. Munroe, Steffen E. Petersen

**Affiliations:** ^1^Barts Heart Centre, Barts Health NHS Trust, London, United Kingdom; ^2^William Harvey Research Institute, NIHR Barts Biomedical Research Centre, Queen Mary University of London, London, United Kingdom

**Keywords:** obesity, lipid profile, mendelian randomization, COVID-19, SARS-CoV-2

## Abstract

**Background:**

Cross-sectional observational studies have reported obesity and cardiometabolic co-morbidities as important predictors of coronavirus disease 2019 (COVID-19) hospitalization. The causal impact of these risk factors is unknown at present.

**Methods:**

We conducted multivariable logistic regression to evaluate the observational associations between obesity traits (body mass index [BMI], waist circumference [WC]), quantitative cardiometabolic parameters (systolic blood pressure [SBP], serum glucose, serum glycated hemoglobin [HbA1c], low-density lipoprotein [LDL] cholesterol, high-density lipoprotein [HDL] cholesterol and triglycerides [TG]) and SARS-CoV-2 positivity in the UK Biobank cohort. One-sample MR was performed by using the genetic risk scores of obesity and cardiometabolic traits constructed from independent datasets and the genotype and phenotype data from the UK Biobank. Two-sample MR was performed using the summary statistics from COVID-19 host genetics initiative. Cox proportional hazard models were fitted to assess the risk conferred by different genetic quintiles of causative exposure traits.

**Results:**

The study comprised 1,211 European participants who were tested positive for severe acute respiratory syndrome coronavirus 2 (SARS-CoV-2) and 387,079 participants who were either untested or tested negative between 16 March 2020 to 31 May 2020. Observationally, higher BMI, WC, HbA1c and lower HDL-cholesterol were associated with higher odds of COVID-19 infection. One-sample MR analyses found causal associations between higher genetically determined BMI and LDL cholesterol and increased risk of COVID-19 (odds ratio [OR]: 1.15, confidence interval [CI]: 1.05–1.26 and OR: 1.58, CI: 1.21–2.06, per 1 standard deviation increment in BMI and LDL cholesterol respectively). Two-sample MR produced concordant results. Cox models indicated that individuals in the higher genetic risk score quintiles of BMI and LDL were more predisposed to COVID-19 (hazard ratio [HR]: 1.24, CI: 1.03–1.49 and HR: 1.37, CI: 1.14–1.65, for the top vs the bottom quintile for BMI and LDL cholesterol, respectively).

**Conclusion:**

We identified causal associations between BMI, LDL cholesterol and susceptibility to COVID-19. In particular, individuals in higher genetic risk categories were predisposed to SARS-CoV-2 infection. These findings support the integration of BMI into the risk assessment of COVID-19 and allude to a potential role of lipid modification in the prevention and treatment.

## Introduction

Severe acute respiratory syndrome coronavirus 2 (SARS-CoV-2), the pathogen of coronavirus disease 2019 (COVID-19), has inflicted a once-in-a-century pandemic. More than 7 million confirmed cases have been reported worldwide with over 407,000 deaths as of June 9 (COVID-19 Map, 2020). Although many countries have navigated past the peak of the epidemic (“flattened the curve”), the viral transmission is expected to continue in the communities for the foreseeable future. Understanding the host factors influencing the disease susceptibility is imperative in unraveling the disease biology and risk stratification. Multiple observational studies have reported that certain patient characteristics (e.g., age, male sex, ethnicity) and comorbid conditions are associated with COVID-19 susceptibility and worse outcomes (Collaborative et al., 2020; Docherty et al., 2020; Li et al., 2020). In particular, elevated body mass index (BMI), a widely used surrogate of obesity, has emerged as an important risk factor for COVID-19 hospital admission, disease severity and in-hospital mortality (Kalligeros et al., 2020; Lighter et al., 2020; Palaiodimos et al., 2020; Simonnet et al., 2020). Likewise, other important constituents of metabolic syndrome namely diabetes and hypertension have been reported to be associated with at least twofold higher risk of severe or fatal COVID-19 (Kumar et al., 2020; Lippi et al., 2020). Nonetheless, given the observational nature of previous COVID-19 studies, the causal impact of obesity and cardiometabolic indices on COVID-19 susceptibility has not been ascertained.

Mendelian randomization (MR) is an analytic technique that uses genetic variants as instruments to estimate the causal effect of an exposure risk factor on an outcome of interest (Smith and Ebrahim, 2003). By leveraging on the Mendel’s law of independent assortment, MR analyses overcome the limitations of confounding, measurement errors and reverse causation frequently encountered in observational studies. Furthermore, it permits causal analysis in the settings where traditional randomized-controlled trials are unfeasible due to ethical or technical reasons. In this study, we examined the causal relationship between obesity traits, quantitative cardiometabolic biomarkers and COVID-19 susceptibility using Mendelian randomization.

## Materials and Methods

### Study Design

We performed multivariable regression analyses of observational data, one-sample MR using individual-level genotype data from the UK Biobank, and two-sample MR using the genome-wide association data provided by COVID-19 host genetics initiative (The COVID-19 Host Genetics Initiative, 2020) to explore if obesity traits (BMI and waist circumference [WC]) and quantitative cardiometabolic parameters (systolic blood pressure [SBP], serum glucose, serum glycated hemoglobin [HbA1c]), low-density lipoprotein [LDL] cholesterol, high-density lipoprotein [HDL] cholesterol and triglycerides [TG]) are causally associated with SARS-CoV-2 positivity (i.e., COVID-19 diagnosis).

### Data Sources

#### UK Biobank

The UK Biobank is a population-based cohort study of 500,000 individuals aged between 40 and 69 years at the time of initial recruitment between 2006 and 2010. It has collected information on health and lifestyle data, physical measurements, biological samples, genotype and multi-modal imaging data (Sudlow et al., 2015). We used the obesity and cardiometabolic measurements taken at the baseline visit where available. Further information on derivation of exposure variables and covariates is available in Supplementary Methods.

From March 16, 2020, the UK Biobank has started releasing COVID-19 test results of the study participants enabled by the Bugbank project (Armstrong et al., 2020; Hilton et al., 2020). We used the data downloaded on June 5, 2020 (the last test date in the sample was May 31, 2020). As the test data was only available for England at the time of analysis, only participants residing in England were included in the analysis. Non-European ancestries were excluded in order to improve homogeneity of the study population and align with the genetic analyses.

This study was covered by the general ethical approval for UK Biobank studies from the NHS National Research Ethics Service on May 10, 2016 (Ref 16/NW/0274). The data underlying this article are available from the UK Biobank^1^.

#### Variant Selection and Genetic Risk Score Construction

We used the variant effect sizes from publicly available genome-wide association meta-analyses conducted in non-UK Biobank samples to avoid circular inferences or overestimation. The following GWAS summary statistics were accessed: BMI and BMI-adjusted WC data from the Genetic Investigation of ANthropometric Traits (GIANT) consortium (Locke et al., 2015; Shungin et al., 2015); fasting serum glucose and HbA1c data from the Meta-Analyses of Glucose and Insulin-related traits Consortium (MAGIC) consortium (Manning et al., 2012; Wheeler et al., 2017); serum LDL cholesterol, HDL cholesterol and TG from the Global Lipids Genetic Consortium (GLGC) (Willer et al., 2013); SBP from Evangelou et al. (2018). Further information on each study was outlined in eMethods. We used the plink (Purcell et al., 2007) software’s “–clump” command with linkage disequilibrium (LD) *r*^2^ threshold of 0.01 to obtain a set of uncorrelated variants at *P* < 5 × 10^–8^. This process produced 77, 75, 250, 26, 45, 101, 125, 73 independent variants for BMI, WC, SBP, serum glucose, HbA1c, LDL cholesterol, HDL cholesterol, triglycerides, respectively (Supplementary Tables 1–8). The weighted genetic risk score (GRS) for each exposure trait was calculated by summing the product of the effect sizes and the number of effect alleles across all selected variants.

### Statistical Analysis

#### Observational Analysis

The participant characteristics stratified by COVID-19 test status are presented as mean ± standard deviation or median and interquartile range (IQR) for continuous variables and number (percentage) for categorical variables. The inter-group differences were compared by unpaired *t*-test, Mann-Whitney *U* test or Fisher’s exact test.

The observational associations between obesity traits, cardiometabolic parameters and SARS-CoV-2 positivity were investigated by multivariable logistic regression adjusted for age at recruitment, sex, multiple deprivation index, smoking history, pre-existing cardiovascular disease, respiratory disease, renal disease and dementia, previous malignancy and exposure-specific additional adjustments. Exposure-specific adjustments were performed to account for confounding by co-existing risk factors while minimizing collinearity. These include hypertension, dyslipidaemia and diabetes for obesity traits; BMI, dyslipidaemia and diabetes for SBP; BMI, hypertension and dyslipidaemia for glycemic traits; BMI, hypertension and diabetes for lipid traits. We considered variance inflation factor (VIF) >3 as an indicator of collinearity between covariates.

#### Mendelian Randomization

We applied the two-stage predictor substitution method using polygenic risk scores for each phenotype data to perform one-sample MR (Burgess, 2013). The one-sample MR analyses were only adjusted for the first 5 principal components to minimize the risk of collider bias. Summary-level genome-wide association meta-analysis data from COVID-19 host genetics initiative (The COVID-19 Host Genetics Initiative, 2020) were used to perform two sample MR. Two-sample MR effect estimates for each exposure trait were calculated by the inverse variance-weighted (IVW) method. Additionally, we used the MR-Egger and weighted median methods to evaluate the validity of genetic instruments (Bowden et al., 2016; Burgess et al., 2016). We assessed the presence of weak instrument bias (also known as violation of *relevance* assumption in MR) by calculating the F-statistic from the linear regression between GRS and measured phenotypes (Davies et al., 2015). Other key assumptions underpinning the MR analysis are: (i) the instrument is independent of confounders (*independence* assumption) and (ii) the instrument exerts its effect exclusively through the risk factor of interest (*exclusion restriction* assumption). We tested these assumptions to a limited extent by adjusting for all potential confounders as utilized in the observational analysis in our MR framework. We assessed the presence of horizontal pleiotropy which would violate the *exclusion restriction* assumption by conducting: (i) the MR-Egger intercept test and (ii) the MR-PRESSO (Verbanck et al., 2018) (Mendelian Randomization Pleiotropy RESidual Sum and Outlier) global test. As a sensitivity analysis, we repeated one-sample MR procedures in a restricted sample of individuals tested for SARS-CoV-2 to explore potential biases introduced by asymptomatic or mildly symptomatic participants who were never tested.

The causal effects were considered only if supported by both one-sample and two-sample MR analyses. The effect sizes are presented by odds ratio (OR) and robust 95% confidence intervals (CI) per 1 standard deviation (SD) change in exposure. We also fitted Cox proportional hazards models adjusted for age at recruitment, sex and the first 5 genetic principal components to evaluate the associations between the weighted GRSs of obesity and cardiometabolic parameters and the risk of SARS-CoV-2 infection. Proportional hazards assumption was checked by assessing the Schoenfeld residuals. All analyses were conducted in the R statistical computing environment (version 3.6.1) (R Core Team, 2016). The two-sample MR analyses were conducted using “MendelianRandomization” (Yavorska and Burgess, 2017) and “TwoSampleMR” (Hemani et al., 2018) R package.

## Results

The clinical characteristics of the study cohort are presented in Table 1. The study comprised 1,211 individuals tested positive for SARS-CoV-2 and 387,079 individuals who were either untested or tested negative between March 16, 2020 and May 31, 2020. Participants with positive test were more likely to be older, male, more deprived and had higher prevalence of cardiometabolic risk factors and comorbidities. The F-statistics of the genetic risk scores were 6491, 664, 7532, 1669, 7816, 26854, 26995, and 19746 for BMI, WC, SBP, serum glucose, HbA1c, LDL cholesterol, HDL cholesterol and triglycerides, respectively. Large F-statistic values (>10) indicated that the MR analyses were unlikely to be affected by the weak instrument bias.

**TABLE 1:** Demographic and clinical characteristics.

Variable	Total	Untested/negative	Positive	*P*
*n*	388,290	387,079	1,211	
Age at recruitment, mean (SD), years	56.6 (8.0)	56.6 (8.0)	57.1 (9.2)	0.049
Male, No. (%)	175,535 (45)	174,895 (45)	640 (53)	<0.001
Multiple deprivation index quintiles, No. (%)				<0.001
1 (most affluent)	75,826 (20)	75,644 (20)	182 (16)	
2	75524 (20)	75,351 (20)	173 (15)	
3	75437 (20)	75,241 (20)	196 (17)	
4	75629 (20)	75,354 (20)	275 (24)	
5 (most deprived)	75513 (20)	75,169 (20)	344 (29)	
Hypertension, No. (%)	130582 (34)	130,034 (34)	548 (45)	<0.001
Dyslipidaemia, No. (%)	114236 (29)	113,801 (29)	435 (36)	<0.001
Diabetes mellitus, No. (%)	27015 (7)	26,835 (7)	180 (15)	<0.001
Smoking status, No. (%)				<0.001
Never	210836 (54)	210,288 (54)	548 (45)	
Previous	139318 (36)	138,795 (36)	523 (43)	
Current	38136 (10)	37,996 (10)	140 (12)	
Cardiovascular disease, No. (%)	35986 (9)	35,789 (9)	197 (16)	<0.001
Respiratory disease, No. (%)	63029 (16)	62,750 (16)	279 (23)	<0.001
Renal disease, No. (%)	15859 (4)	15,735 (4)	124 (10)	<0.001
Previous malignancy, No. (%)	67852 (18)	67,618 (18)	234 (19)	0.097
Dementia, No. (%)	1317 (0.3)	1,270 (0.3)	47 (4)	<0.001
Body mass index, mean (SD), kg/m^2^	27.3 (4.7)	27.3 (4.7)	28.7 (5.4)	<0.001
Waist circumference, mean (SD), cm	90.1 (13.4)	90.1 (13.4)	94.3 (14.4)	<0.001
Systolic blood pressure, mean (SD), mmHg	141.0 (20.5)	141.0 (20.5)	143.1 (21.8)	<0.001
Fasting glucose, median [IQR], mmol/L	4.9 [4.6, 5.3]	4.9 [4.6, 5.3]	5.0 [4.6, 5.4]	0.221
HbA1c, median [IQR], mmol/mol	35.1 [32.7, 37.6]	35.1 [32.7, 37.6]	35.6 [33.1, 38.7]	<0.001
LDL cholesterol, mean (SD), mmol/L	3.6 (0.9)	3.6 (0.9)	3.5 (0.9)	<0.001
HDL cholesterol, mean (SD), mmol/L	1.5 (0.4)	1.5 (0.4)	1.4 (0.3)	<0.001
Triglycerides, mean (SD), mmol/L	1.7 (1.0)	1.7 (1.0)	1.9 (1.1)	<0.001

*HbA1c, glycated hemoglobin; LDL, low-density lipoprotein; HDL, high-density lipoprotein; SD, standard deviation; IQR, interquartile range.*

### Relationship Between Obesity Phenotypes and COVID-19 Susceptibility

In observational analyses, higher BMI and WC were associated with higher odds of SARS-CoV-2 positivity (OR: 1.13 [CI: 1.07–1.20], OR: 1.15 [CI: 1.08–1.23], for 1 SD increment in BMI and WC, respectively) (Table 2). One-sample MR analyses indicated a causal relationship between higher BMI and increased odds of positive test (OR: 1.15 [CI: 1.05–1.26]) but WC was not causally associated with COVID-19 status. The MR analyses additionally controlled for other covariates included in the observational analyses did not attenuate the findings (Supplementary Table 9). Two sample MR by IVW method confirmed the causal association between BMI and COVID-19 identified in one-sample analysis (Table 3 and Supplementary Figure 1). Analyses by MR-Egger and weighted median methods produced directionally concordant results (Supplementary Table 10). There was no evidence of substantial horizontal pleiotropy as indicated by Egger intercept test and MR-PRESSO global test (Egger intercept *P* = 0.459, MR-PRESSO *P* = 0.557). Sensitivity analyses in a restricted cohort of individuals tested for SARS-CoV-2 (positive = 1,211, negative = 4,086) produced an MR estimate for BMI in a consistent effect direction compared to the main results (Supplementary Table 11).

**TABLE 2:** Observational and one-sample MR analysis.

Trait	Observational effect size	Observational 95% CI	Observational *P*-value	MR effect size	MR 95% CI	MR *P*-value
BMI	1.13	1.07–1.20	1.26 × 10^–5^	1.15	1.05–1.26	0.003
WC	1.15	1.08–1.23	1.91 × 10^–5^	1.06	0.95–1.18	0.270
SBP	0.99	0.93–1.06	0.863	1.02	1.00–1.04	0.111
Fasting glucose	1.04	0.99–1.10	0.090	0.73	0.33–1.61	0.434
HbA1c	1.04	1.004–1.09	0.031	0.98	0.92–1.04	0.519
LDL cholesterol	0.97	0.91–1.03	0.335	1.58	1.21–2.06	0.001
HDL cholesterol	0.85	0.78–0.92	3.17 × 10^–5^	1.03	0.57–1.85	0.918
Triglycerides	0.99	0.93–1.05	0.748	1.17	0.90–1.51	0.243

*Effect sizes represent odds ratio for every 1 standard deviation increment in exposure trait. The standard deviations are: 4.7 kg/m^2^, 13.4 cm, 20.5 mmHg, 1.2 mmol/L, 6.3 mmol/mol, 0.87 mmol/L, 0.38 mmol/L and 1.0 mmol/L, for BMI, WC, SBP, fasting glucose, HbA1c, LDL cholesterol, HDL cholesterol and triglycerides, respectively. BMI, body mass index; WC, waist circumference; SBP, systolic blood pressure, HbA1c, glycated hemoglobin; LDL, low-density lipoprotein; HDL, high-density lipoprotein; CI, confidence interval; MR, Mendelian randomization.*

**TABLE 3:** Two-sample MR analysis.

Trait	IVW effect size	IVW 95% CI	IVW *P*-value	Egger intercept	Egger intercept *P*-value	MR-PRESSO global test *P*-value
BMI	1.80	1.28–2.54	0.001	0.013	0.129	0.557
WC	1.25	0.86–1.79	0.238	0.004	0.772	0.817
SBP	0.79	0.52–1.20	0.278	0.00003	0.966	0.674
Fasting glucose	0.78	0.51–1.19	0.253	−0.019	0.111	0.791
HbA1c	0.26	0.01–8.47	0.446	0.022	0.024	0.813
LDL cholesterol	1.20	1.06–1.37	0.006	0.001	0.899	0.741
HDL cholesterol	1.03	0.84–1.25	0.803	−0.01	0.144	0.875
Triglycerides	1.10	0.79–1.53	0.578	0.002	0.876	0.939

*Effect sizes represent odds ratio for every 1 standard deviation increment in exposure trait. BMI, body mass index; WC, waist circumference; SBP, systolic blood pressure, HbA1c, glycated hemoglobin; LDL, low-density lipoprotein; HDL, high-density lipoprotein; IVW, inverse variance weighted; CI, confidence interval; MR, Mendelian randomization.*

### Relationship Cardiometabolic Indices and COVID-19 Susceptibility

The observational analysis indicated that higher HbA1c and lower serum HDL-cholesterol were associated with higher odds of SARS-CoV-2 positive result (OR: 1.04 [CI: 1.004–1.09]; OR: 0.85 [CI: 0.78–0.92] for 1 SD increment in HbA1c and HDL cholesterol, respectively) (Table 2). After adjusting for potential confounders, SBP, fasting glucose, LDL-cholesterol and triglycerides were not associated with COVID-19 status. In contrast, in one-sample MR analysis, higher LDL cholesterol was associated with higher odds of SARS-CoV-2 infection (OR: 1.58 [CI: 1.21–2.06]). No other causal relationship was identified between other cardiometabolic parameters and COVID-19 status. Additional adjustment of one-sample MR with observation covariates yielded similar results (Supplementary Table 9). These findings were also supported by two-sample MR results (Table 3). There was no evidence of horizonal pleiotropy (Egger intercept *P* = 0.899, MR-PRESSO *P* = 0.741). The MR analysis in a restricted sample of individuals who had received testing supported the causal association between LDL cholesterol and COVID-19 susceptibility (OR: 1.74 [CI: 1.22–12.47]).

### Polygenic Prediction of COVID-19 Risk

**FIGURE 1:**
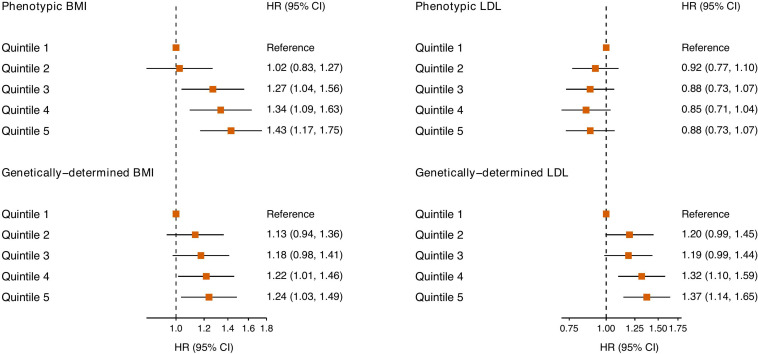
Association between measured and genetic BMI, LDL cholesterol, and SARS-COV-2 infection. SARS-COV-2, severe acute respiratory syndrome coronavirus 2; BMI, body mass index; LDL, low-density lipoprotein; HR, hazard ratio; CI, confidence interval.

Genetically determined BMI was associated with higher risk of SARS-CoV-2 infection with the greatest risk observed in the top quintile of BMI-GRS (Hazard ratio [HR]: 1.24 [CI: 1.03–1.49] compared to the lowest quintile) (Figure 1). Likewise, genetically higher exposure to LDL cholesterol was related to increased risk of COVID-19 (HR: 1.37 [CI: 1.14–1.65] for the top quintile vs the bottom quintile). Observationally, the same pattern of relationship was identified only for BMI.

## Discussion

In this study, we examined the causal relationship between obesity traits, quantitative cardiometabolic biomarkers and COVID-19 susceptibility using Mendelian randomization. We identified a causal relationship between BMI and LDL cholesterol and susceptibility to COVID-19. In contrast, WC and other quantitative cardiometabolic markers such as SBP, serum glucose, HbA1c, HDL and triglycerides, were not causally linked to increased risk of SARS-CoV-2 infection. The highest risk was observed in individuals belonging to the top genetic risk quintiles of BMI and LDL cholesterol.

Recent observational data from China have identified that age and obesity related complications such as hypertension and type 2 diabetes mellitus may be risk factors for development of severe COVID-19 (Shi et al., 2020). A high prevalence of obesity in severe COVID-19 patients has also been observed in recent studies (Caussy et al., 2020; Lighter et al., 2020; Simonnet et al., 2020), with greater requirement for mechanical ventilation in those who are in the overweight or obese categories. However, these purely observational studies were open to confounding despite attempts at adjustments for potential known or measured factors. Our study attempted to overcome the inherent limitations of observational data by instrumental variable analysis using genetic variants which are randomly allocated at conception. This approach provided the evidence for causal linkage between obesity and COVID-19 diagnosis while circumventing the issues related to confounding, recall bias, measurement errors and reverse causation.

There are a number of possible explanations for our findings. Obesity has been associated with more severe and prolonged disease as noted with previous epidemics (Huttunen and Syrjänen, 2013; Honce and Schultz-Cherry, 2019). Obese patients are more likely to have reduced and restrictive pulmonary function, which can subsequently improve following weight reduction through bariatric surgery (Copley et al., 2020). The immune response may be adversely altered in obesity which could have deleterious effect on the respiratory function. In particular interleukin-6 levels and type 2 inflammation have been shown to be increased in patients with obesity and metabolic syndrome, along with abnormal secretion of adipokines and cytokines such as interferon and TNF-a in individuals with abdominal obesity (Huttunen and Syrjänen, 2013; Peters et al., 2016; Zhang et al., 2017).

After controlling for potential confounders, LDL cholesterol concentration was not found to be associated with COVID-19 in our observational analysis. This finding is contrary to recent observational studies. A retrospective study from Wuhan, China, of SARS-CoV-2 infected patients found that total cholesterol and LDL levels were inversely associated with disease severity (Wei et al., 2020). A different group from Wenzhou, China also suggest in an observational study that those with the infection had significantly lower total cholesterol, HDL and LDL (Hu et al., 2020). It should be noted that both studies reported cross-sectional inter-group differences without robust adjustment for potential confounders. Conversely, a recent study investigating the association between LDL cholesterol and risk of sepsis in 3,961 patients admitted to hospital with a serious infection found that measured LDL levels were associated with increased risk of sepsis and admission to the intensive care unit (Feng et al., 2019). However, after adjusting for clinical factors and re-analysis in a genetic model, the association was no longer present, suggesting that comorbidities accounted for the observations seen in the unadjusted raw measures. This supports the findings of our study which showed discordance between the trend seen in the purely observational LDL levels compared to the findings from the MR analysis, which is less prone to confounding factors. Furthermore, it should be noted that the MR effect size represents a lifelong exposure in contrast to cross-sectionally measured LDL cholesterol which provides a snapshot information influenced by intercurrent illness and medical therapy.

Lipid metabolism plays a pivotal role in viral lifecycle including replication, membrane homeostasis, endocytosis and exocytosis (Abu-Farha et al., 2020). Indeed, previous experience from SARS-CoV-1 infection indicated altered lipid metabolism following recovery, suggesting a biological relationship (Wu et al., 2017). Cholesterol depletion by drug treatment had been shown to suppress an avian coronavirus (infectious bronchitis virus) by disrupting the lipid rafts which enable cellular entry (Guo et al., 2017). Another study investigating porcine delta coronavirus found that pharmacological sequestration effectively blocked viral attachment and internalization (Jeon and Lee, 2018). These studies together with our own study require further exploration and research assessing the potential implications of LDL modification, such as with the use of statin medications, for reducing susceptibility of developing COVID-19 or its severity.

Most previous observational studies have reported the predictors of COVID-19 severity rather than COVID-19 susceptibility. One study (Gu et al., 2020) that investigated the relationships between the clinical risk factors and COVID-19 susceptibility found positive associations between pre-existing respiratory disease (OR: 3.28 [2.71–3.97]), circulatory disease (OR: 2.46 [2.02–2.99]), type 2 diabetes (OR: 1.92 [1.53–2.41]), chronic kidney disease (OR: 2.58 [1.98–3.37]), liver disease (OR: 3.02 [2.18–4.17]) and autoimmune disease (OR: 2.55 [2.02–3.21]). While the effect sizes of the intermediate phenotypes investigated in our study are generally smaller, it is perhaps unsurprising given that diseases usually represent extreme phenotypes.

### Clinical Relevance

The implications of our findings are likely to be significant in forming a risk assessment tool for those who are admitted to hospital with COVID-19 and who may require more intense monitoring and/or escalation of treatment at an earlier stage. A recently reported COVID-19 risk assessment tool for severe disease or mortality (Jankowski et al., 2020) included BMI ≥ 35 kg/m^2^ as an independent risk factor. Our data supports this but also highlights that the association between BMI and COVID-19 susceptibility is monotonous. Although the risk is highest for individuals in the highest BMI category, those with moderately elevated BMI still have non-negligible risk of COVID-19 even after accounting for co-morbidities. Our findings may have an impact on public health policy, whereby those who fall in the at-risk obese category or those with extreme hyperlipidemia in the general population may require more rigorous social distancing or shielding, particularly if a second wave or future pandemics becomes a reality. Studies assessing the role for cholesterol modification therapy during illness or hospital admission could be undertaken to assess potential impact on outcomes (Greenhalgh et al., 2020). Longer term and wider emphasis on tackling obesity and dyslipidaemia, which already features in many cardiovascular disease risk algorithms (Khanji et al., 2016), through lifestyle advice and interventions should be made a priority as part of a prevention strategy (Khanji et al., 2018).

### Strengths and Limitations

We believe this is the first study to use MR to evaluate causality of obesity and cardiometabolic traits in the context of COVID-19 susceptibility. The depth of data means that most potential confounders and cardiometabolic parameters can be assessed in a robust manner. We used large independent datasets to obtain the effect estimates for genetic risk scores which mitigated the risks of circular inferences or overestimation in our results. The one-sample MR findings were corroborated by two-sample MR, providing an additional line of evidence.

There are certain limitations to our study. First, due to the design of genetic risk scores constructed from predominantly European discovery analyses and a small number of SARS-CoV-2 positive minority ethnicities in the COVID-19 dataset (*n* = 197), we confined our analyses to Caucasians which limits the generalizability of our findings across other ethnicities. This is especially important due to the disproportionate impact of COVID-19 on non-White individuals. Future MR studies should investigate the influence of cardiometabolic risk factors on COVID-19 in populations of African and other ancestries to better inform the public health policies. Second, as the UK Biobank is a major contributor (∼56%) of COVID-19 host genetics initiative, the data used in one-sample and two-sample MR are not entirely independent and the findings of our study need to be confirmed with an independent validation dataset. Third, although we have been able to assess causality for COVID-19 diagnosis based on the available data, future analysis with additional test result data along with detailed information on severity (such ventilation requirement, thromboembolic complications) will be crucial to contextualize our findings. With the on-going enrichment of UK Biobank COVID-19 database alongside the information provided by global consortia, it will soon be possible to dissect these questions in a hypothesis-driven manner.

## Conclusion

This is the first study to identify the causal relationships between BMI, LDL cholesterol and susceptibility to SARS-CoV-2 infection. We found that the individuals in the top quintiles of genetically determined BMI and LDL are especially vulnerable. Altogether, our findings suggest that BMI should be considered integral in the future risk assessment for COVID-19. The influence of lipid metabolism on virus proliferation and the role of LDL lowering medications for prophylaxis and treatment of SARS-CoV-2 should be investigated in the future studies.

## Data Availability Statement

The datasets presented in this study can be found in online repositories. The names of the repository/repositories and accession number(s) can be found in the article/Supplementary Material.

## Ethics Statement

The studies involving human participants were reviewed and approved by This study was covered by the general ethical approval for UK Biobank studies from the NHS National Research Ethics Service on 10th May 2016 (Ref 16/NW/0274). The patients/participants provided their written informed consent to participate in this study.

## Author Contributions

NA conceived the hypothesis, preformed data analysis, and wrote the manuscript. MK wrote and reviewed the manuscript. PM and SP reviewed the manuscript and provided edits. All authors contributed to the article and approved the submitted version.

## Conflict of Interest

SP provides consultancy to and is shareholder of Circle Cardiovascular Imaging Inc., Calgary, Canada. The remaining authors declare that the research was conducted in the absence of any commercial or financial relationships that could be construed as a potential conflict of interest.

## Footnotes

1 http://www.ukbiobank.ac.uk/register-apply/
