# Supplementary material for: Causal Inference for Genetic Obesity, Cardiometabolic Profile and COVID-19 Susceptibility: A Mendelian Randomization Study
Source: Front Genet. 2020 Nov 11;11:586308. doi: 10.3389/fgene.2020.586308 (PMC7686798; doi:10.3389/fgene.2020.586308)
Supplement: Supplementary file 1 [file Data_Sheet_1.DOCX]

**Supplementary Materials**

**Supplementary Methods**

**Derivation of exposure variables and covariates**

Body mass index was calculated by dividing weight (kg) by height (m)^2^. We calculated the average of multiple systolic blood pressure (SBP) measurements taken by Omron 705 IT electronic blood pressure monitor and manual sphygmomanometer. The averaged SBP was then corrected for anti-hypertensive medication use by addition of 15mmHg^1^. Serum glucose and triglycerides were measured by an enzymatic method. Direct low-density lipoprotein (LDL) cholesterol serum concentration was measured by enzymatic selective protection method and high-density lipoprotein (HDL) cholesterol was measured by enzyme immuno-inhibition method. All biochemistry tests were performed on a Beckman Coulter AU5800 clinical chemistry analyser (Beckman Coulter, Brea, California, USA). Missing direct LDL cholesterol measurements were replaced by the estimates derived by the Friedewald equation if serum triglycerides concentration was ≤4 mmol/L^2^. We adjusted for the use of lipid-lowering medications by multiplying the LDL cholesterol values with 1.43 to approximate untreated LDL cholesterol serum concentration^3^. Serum glycated haemoglobin (HbA1c) assay was performed using the Bio-Rad Variant II Turbo analysers. Diabetes mellitus, hypertension, dyslipidaemia, cardiovascular disease, respiratory disease, renal disease, dementia and cancer diagnoses were ascertained from a combination of participants’ response to the health questionnaire, verbal interview at the UK Biobank assessment centre, the hospital episodes data and cancer registry. To identify genetically determined European ethnicity, we first defined ancestry clusters by applying k-means clustering algorithm with k=4 on genotypic principal component 1 and 2 (PC 1 and PC 2) separately. The number of clusters (k) was chosen as 4 to represent the 4 main ethnic groups within the UK Biobank: White, African, Asian and Chinese. An overall clustering was carried out by intersection of PC1-4means-clusters and PC2-4means-clusters. The largest overlapping cluster represents the European (White) ancestry while discordant clustering between PC1and PC2 represents ‘Mixed/Other’ category. European ancestry was ascertained only if the self-reported ethnicity agreed with k-means clustering results. Genotypes directly-called by two closely related UK BiLEVE Axiom and UK Biobank Axiom microarrays (Affymetrix) were imputed using the Haplotype Reference Consortium and merged UK10K and 1000 Genomes phase 3 reference panels^4^.

**Genome-wide association summary statistics**

We obtained the summary statistics of BMI and BMI-adjusted waist circumference (WC) data from the Genetic Investigation of ANthropometric Traits (GIANT) consortium^5,6^. The BMI meta-analysis included up to 339,224 individuals of predominantly European descent from 125 studies. Age, age^2^ and other study-specific covariates were regressed out from BMI and the residuals were inverse normal transformed prior to association analyses. Residuals were calculated separately by sex and case/control status for studies with related individuals while sex was used as an additional covariate in family studies. The causal estimates for the two-sample Mendelian randomisation (MR) were converted to raw BMI units (kg/m^2^) by assuming a median BMI standard deviation (SD) of 4.5 kg/m^2^. The WC meta-analysis comprised 224,459 participants (94% European). WC was additionally adjusted for BMI, but otherwise transformed in the same way as BMI analyses. We assumed a median WC SD of 11.9 cm for conversion of transformed effect sizes to raw WC units (cm). SBP summary statistics were obtained from the study conducted by Evangelou et al^7^ (maximum sample size = 1,006,863 European individuals). The discovery analysis of SBP was adjusted for sex, age, age^2^, BMI and other study-specific covariates. We used the variant effect sizes from non-UK Biobank sample (beta coefficients from the International Consortium of Blood Pressure [ICBP] discovery meta-analysis or from the replication meta-analysis) to obtain unbiased estimates. The summary data for fasting serum glucose and HbA1c data were retrieved from the Meta-Analyses of Glucose and Insulin-related traits Consortium (MAGIC) consortium^8,9^. Fasting glucose analysis was adjusted for age, sex and study-level covariates in a sample of up to 58,074 non-diabetic European individuals. We used serum HbA1c summary statistics derived from the analyses adjusted for age, sex and study-specific covariates in the European cohort (n = 123,665). For the lipid traits, we accessed the data from the Global Lipids Genetic Consortium (GLGC)^10^. Genome-wide discovery analyses for fasting lipid parameters (LDL cholesterol, HDL cholesterol and triglycerides) were performed in up to 188,577 European individuals. The residuals of lipid traits adjusted for age, age^2^, sex and study-specific covariates were inverse normal transformed. Therefore, the two-sample MR estimates represent the median SD values of lipid traits (0.96 mmol/L, 0.38 mmol/L and 0.98 mmol/L for LDL cholesterol, HDL cholesterol and triglycerides, respectively). For the summary statistics of COVID-19 susceptibility, we downloaded the second round meta-analysis data released on May 15 2020 from COVID-19 host genetics initiative^11^ (<https://www.covid19hg.org/>). We used the COVID-19 phenotype as defined in analysis 5 (ANA5) which considered individuals diagnosed with COVID-19 as cases and those without documented diagnosis of COVID-19 as controls (n cases = 1,678, n controls = 674,635). The participating cohorts are BioMe (n cases = 20, n controls = 10,169), FinnGen (n cases = 209, n controls = 203,431), Genes & Health (n cases = 64, n controls = 27,351), Lifelines CytoSNP (n cases = 62, n controls = 5,750), Lifelines Global Screening Array (n cases = 189, n controls = 15,975), Netherlands Twin Registry (n cases = 163, n controls = 3,160), Partners Healthcare Biobank (n cases = 108, n controls = 31,119) and UK Biobank (n cases = 863, n controls = 377,480). The analysis model was adjusted for age, age^2^, sex, age x sex, genetic principle components and study-specific covariates.

**Supplementary Tables**

**Supplementary Table S1. Genetic variants for body mass index**

| **rsID** | **EA** | **NEA** | **Effect size** | **Standard error** | **P** |
| --- | --- | --- | --- | --- | --- |
| rs1558902 | A | T | 0.0818 | 0.0031 | 7.5E-153 |
| rs6567160 | C | T | 0.0556 | 0.0036 | 3.93E-53 |
| rs13021737 | G | A | 0.0601 | 0.004 | 1.11E-50 |
| rs10938397 | G | A | 0.0402 | 0.0031 | 3.21E-38 |
| rs543874 | G | A | 0.0482 | 0.0039 | 2.62E-35 |
| rs2207139 | G | A | 0.0447 | 0.004 | 4.13E-29 |
| rs11030104 | A | G | 0.0414 | 0.0038 | 5.56E-28 |
| rs3101336 | C | T | 0.0334 | 0.0031 | 2.66E-26 |
| rs7138803 | A | G | 0.0315 | 0.0031 | 8.15E-24 |
| rs10182181 | G | A | 0.0307 | 0.0031 | 8.78E-24 |
| rs3888190 | A | C | 0.0309 | 0.0031 | 3.14E-23 |
| rs1516725 | C | T | 0.0451 | 0.0046 | 1.89E-22 |
| rs12446632 | G | A | 0.0403 | 0.0046 | 1.48E-18 |
| rs2287019 | C | T | 0.036 | 0.0042 | 4.59E-18 |
| rs16951275 | T | C | 0.0311 | 0.0037 | 1.91E-17 |
| rs3817334 | T | C | 0.0262 | 0.0031 | 5.15E-17 |
| rs2112347 | T | G | 0.0261 | 0.0031 | 6.19E-17 |
| rs12566985 | G | A | 0.0242 | 0.0031 | 3.28E-15 |
| rs3810291 | A | G | 0.0283 | 0.0036 | 4.81E-15 |
| rs7141420 | T | C | 0.0235 | 0.0031 | 1.23E-14 |
| rs13078960 | G | T | 0.0297 | 0.0039 | 1.74E-14 |
| rs10968576 | G | A | 0.0249 | 0.0033 | 6.61E-14 |
| rs17024393 | C | T | 0.0658 | 0.0088 | 7.03E-14 |
| rs657452 | A | G | 0.0227 | 0.0031 | 5.48E-13 |
| rs12429545 | A | G | 0.0334 | 0.0047 | 1.09E-12 |
| rs12286929 | G | A | 0.0217 | 0.0031 | 1.31E-12 |
| rs13107325 | T | C | 0.0477 | 0.0068 | 1.83E-12 |
| rs11165643 | T | C | 0.0218 | 0.0031 | 2.07E-12 |
| rs7903146 | C | T | 0.0234 | 0.0034 | 1.11E-11 |
| rs10132280 | C | A | 0.023 | 0.0034 | 1.14E-11 |
| rs17405819 | T | C | 0.0224 | 0.0033 | 2.07E-11 |
| rs1016287 | T | C | 0.0229 | 0.0034 | 2.25E-11 |
| rs4256980 | G | C | 0.0209 | 0.0031 | 2.9E-11 |
| rs17094222 | C | T | 0.0249 | 0.0038 | 5.94E-11 |
| rs12401738 | A | G | 0.0211 | 0.0033 | 1.15E-10 |
| rs7599312 | G | A | 0.022 | 0.0034 | 1.17E-10 |
| rs2365389 | C | T | 0.02 | 0.0031 | 1.63E-10 |
| rs205262 | G | A | 0.0221 | 0.0035 | 1.75E-10 |
| rs2820292 | C | A | 0.0195 | 0.0031 | 1.83E-10 |
| rs12885454 | C | A | 0.0207 | 0.0033 | 1.94E-10 |
| rs9581854 | T | C | 0.0298 | 0.0047 | 2.29E-10 |
| rs16851483 | T | G | 0.0483 | 0.0077 | 3.55E-10 |
| rs1167827 | G | A | 0.0202 | 0.0033 | 6.33E-10 |
| rs758747 | T | C | 0.0225 | 0.0037 | 7.47E-10 |
| rs1928295 | T | C | 0.0188 | 0.0031 | 7.91E-10 |
| rs9925964 | A | G | 0.0192 | 0.0031 | 8.11E-10 |
| rs11126666 | A | G | 0.0207 | 0.0034 | 1.33E-09 |
| rs2650492 | A | G | 0.0207 | 0.0035 | 1.92E-09 |
| rs6804842 | G | A | 0.0185 | 0.0031 | 2.48E-09 |
| rs12940622 | G | A | 0.0182 | 0.0031 | 2.49E-09 |
| rs11847697 | T | C | 0.0492 | 0.0084 | 3.99E-09 |
| rs4740619 | T | C | 0.0179 | 0.0031 | 4.56E-09 |
| rs13191362 | A | G | 0.0277 | 0.0048 | 7.34E-09 |
| rs3736485 | A | G | 0.0176 | 0.0031 | 7.41E-09 |
| rs17001654 | G | C | 0.0306 | 0.0053 | 7.76E-09 |
| rs11191560 | C | T | 0.0308 | 0.0053 | 8.45E-09 |
| rs1528435 | T | C | 0.0178 | 0.0031 | 1.2E-08 |
| rs2075650 | A | G | 0.0258 | 0.0045 | 1.25E-08 |
| rs1000940 | G | A | 0.0192 | 0.0034 | 1.28E-08 |
| rs2033529 | G | A | 0.019 | 0.0033 | 1.39E-08 |
| rs11583200 | C | T | 0.0177 | 0.0031 | 1.48E-08 |
| rs9400239 | C | T | 0.0188 | 0.0033 | 1.61E-08 |
| rs10733682 | A | G | 0.0174 | 0.0031 | 1.83E-08 |
| rs11688816 | G | A | 0.0172 | 0.0031 | 1.89E-08 |
| rs11057405 | G | A | 0.0307 | 0.0055 | 2.02E-08 |
| rs2121279 | T | C | 0.0245 | 0.0044 | 2.31E-08 |
| rs29941 | G | A | 0.0182 | 0.0033 | 2.41E-08 |
| rs11727676 | T | C | 0.0358 | 0.0064 | 2.55E-08 |
| rs3849570 | A | C | 0.0188 | 0.0034 | 2.6E-08 |
| rs6477694 | C | T | 0.0174 | 0.0031 | 2.67E-08 |
| rs7899106 | G | A | 0.0395 | 0.0071 | 2.96E-08 |
| rs2176598 | T | C | 0.0198 | 0.0036 | 2.97E-08 |
| rs2245368 | C | T | 0.0317 | 0.0057 | 3.19E-08 |
| rs17724992 | A | G | 0.0194 | 0.0035 | 3.42E-08 |
| rs7243357 | T | G | 0.0217 | 0.004 | 3.86E-08 |
| rs1808579 | C | T | 0.0167 | 0.0031 | 4.17E-08 |
| rs2033732 | C | T | 0.0192 | 0.0035 | 4.89E-08 |

EA, effect allele; NEA, non-effect allele

**Supplementary Table S2. Genetic variants for waist circumference**

| **rsID** | **EA** | **NEA** | **Effect size** | **Standard error** | **P** |
| --- | --- | --- | --- | --- | --- |
| rs606452 | A | C | 0.028 | 0.0048 | 1.1E-08 |
| rs9864077 | T | C | 0.022 | 0.0037 | 1.3E-09 |
| rs798489 | C | T | 0.025 | 0.0037 | 1.3E-11 |
| rs17451107 | T | C | 0.026 | 0.0036 | 1.3E-13 |
| rs395962 | T | G | 0.029 | 0.0036 | 1.3E-15 |
| rs13210323 | A | C | 0.022 | 0.0038 | 1.4E-08 |
| rs12608504 | A | G | 0.02 | 0.0036 | 1.5E-08 |
| rs984222 | C | G | -0.036 | 0.0035 | 1.5E-25 |
| rs11144688 | G | A | 0.034 | 0.006 | 1.9E-08 |
| rs757608 | A | G | 0.027 | 0.0036 | 1E-13 |
| rs3791679 | A | G | 0.035 | 0.0039 | 2.1E-19 |
| rs16957304 | A | G | 0.059 | 0.011 | 2.5E-08 |
| rs2179129 | A | G | 0.019 | 0.0034 | 2.6E-08 |
| rs1879529 | G | T | 0.024 | 0.0038 | 2.9E-10 |
| rs12656497 | T | C | 0.022 | 0.0034 | 2E-10 |
| rs12330322 | C | T | 0.022 | 0.004 | 3.2E-08 |
| rs13083798 | A | G | 0.02 | 0.0034 | 3.4E-09 |
| rs7970350 | C | T | 0.019 | 0.0034 | 3.8E-08 |
| rs473902 | T | G | 0.049 | 0.0071 | 4.3E-12 |
| rs1344674 | A | G | -0.024 | 0.0033 | 4.3E-13 |
| rs2160077 | G | A | 0.018 | 0.0033 | 4.5E-08 |
| rs2047937 | C | T | 0.019 | 0.0034 | 4.7E-08 |
| rs849140 | T | C | 0.029 | 0.0034 | 4.7E-17 |
| rs1812175 | A | G | -0.033 | 0.0045 | 4E-13 |
| rs979012 | T | C | 0.033 | 0.0036 | 5.4E-20 |
| rs1776897 | G | T | 0.061 | 0.0067 | 5.6E-20 |
| rs12317176 | T | C | 0.02 | 0.0035 | 5.9E-09 |
| rs12991495 | T | C | 0.028 | 0.0037 | 6.2E-14 |
| rs4886782 | G | A | 0.024 | 0.0036 | 6E-12 |
| rs4567683 | A | G | 0.022 | 0.0038 | 7.7E-09 |
| rs459193 | A | G | 0.025 | 0.0038 | 7.7E-11 |
| rs12493901 | G | A | 0.021 | 0.0034 | 8.3E-10 |
| rs7621331 | A | G | 0.021 | 0.0036 | 9.4E-09 |
| rs3760318 | G | A | 0.021 | 0.0035 | 9E-10 |
| rs991967 | C | A | 0.026 | 0.0037 | 1.1E-12 |
| rs7536458 | G | T | -0.03 | 0.0038 | 1.2E-15 |
| rs1784203 | G | A | -0.031 | 0.0054 | 1.3E-08 |
| rs12679556 | G | T | 0.026 | 0.0039 | 1.3E-11 |
| rs11205277 | G | A | 0.027 | 0.0036 | 1.3E-13 |
| rs6715793 | T | C | 0.019 | 0.0034 | 1.4E-08 |
| rs2052670 | G | A | 0.02 | 0.0035 | 1.5E-08 |
| rs4542783 | C | T | -0.023 | 0.004 | 1.7E-08 |
| rs2274432 | A | G | 0.025 | 0.0036 | 1.7E-12 |
| rs780159 | G | A | 0.021 | 0.0035 | 1.8E-09 |
| rs6772896 | T | C | 0.024 | 0.0036 | 1.8E-11 |
| rs6556301 | T | G | 0.028 | 0.0039 | 1.8E-12 |
| rs806794 | G | A | -0.03 | 0.0037 | 1.9E-15 |
| rs4239436 | G | A | 0.04 | 0.0041 | 1E-22 |
| rs7166081 | A | G | 0.024 | 0.0039 | 2.1E-09 |
| rs72961013 | A | G | 0.076 | 0.011 | 2.1E-11 |
| rs2071449 | A | C | 0.032 | 0.0036 | 2.5E-18 |
| rs4868125 | G | C | 0.021 | 0.0036 | 2.9E-09 |
| rs10041657 | A | G | 0.025 | 0.004 | 2.9E-10 |
| rs12207675 | C | T | 0.031 | 0.0052 | 3.1E-09 |
| rs10748826 | C | T | -0.023 | 0.0037 | 3.4E-10 |
| rs4141278 | C | T | 0.034 | 0.0043 | 3.4E-15 |
| rs822531 | T | C | 0.024 | 0.0044 | 3.7E-08 |
| rs2214442 | G | A | 0.026 | 0.0045 | 3.9E-09 |
| rs9435732 | T | C | -0.031 | 0.0038 | 4.1E-16 |
| rs7684221 | A | G | -0.026 | 0.0047 | 4.2E-08 |
| rs9977276 | G | T | 0.022 | 0.004 | 4.4E-08 |
| rs7854560 | T | C | 0.026 | 0.0037 | 4.8E-12 |
| rs4246302 | G | A | 0.022 | 0.0037 | 5.7E-09 |
| rs9389986 | A | T | -0.024 | 0.0037 | 5.7E-11 |
| rs998584 | A | C | 0.029 | 0.0038 | 6.4E-15 |
| rs2638953 | C | G | 0.024 | 0.0036 | 6.5E-11 |
| rs272869 | G | A | 0.021 | 0.0034 | 6.7E-10 |
| rs2124969 | C | T | 0.02 | 0.0034 | 7.1E-09 |
| rs12127195 | A | G | 0.021 | 0.0037 | 7.7E-09 |
| rs2745353 | T | C | 0.029 | 0.0033 | 7.9E-19 |
| rs10516107 | A | G | 0.023 | 0.0036 | 8.3E-11 |
| rs710841 | T | C | 0.029 | 0.0038 | 8.5E-14 |
| rs3786897 | G | A | 0.02 | 0.0035 | 8.8E-09 |
| rs7801581 | T | C | 0.027 | 0.0042 | 8E-11 |
| rs7162542 | G | C | 0.038 | 0.0034 | 9.7E-29 |

EA, effect allele; NEA, non-effect allele

**Supplementary Table S3. Genetic variants for systolic blood pressure**

| **rsID** | **EA** | **NEA** | **Effect size** | **Standard error** | **P*** |
| --- | --- | --- | --- | --- | --- |
| rs880315 | T | C | -0.5218 | 0.0499 | 9.59E-56 |
| rs17367504 | A | G | 0.7774 | 0.0639 | 1.6E-104 |
| rs3820068 | A | G | 0.3361 | 0.0596 | 3.31E-14 |
| rs2807337 | T | C | 0.1551 | 0.0423 | 2.78E-09 |
| rs79598313 | T | C | 0.456 | 0.1367 | 2.4E-11 |
| rs3737801 | C | G | 0.4246 | 0.0954 | 1.02E-10 |
| rs11210029 | A | G | -0.1608 | 0.0476 | 8.92E-11 |
| rs7515635 | T | C | 0.2382 | 0.0463 | 1.25E-16 |
| rs839755 | A | C | -0.1499 | 0.0434 | 5.41E-18 |
| rs11579440 | T | C | 0.2794 | 0.0653 | 3.24E-10 |
| rs10923038 | A | C | 0.1279 | 0.0481 | 1.36E-10 |
| rs10922502 | A | G | -0.2283 | 0.0483 | 6.14E-20 |
| rs7514579 | A | C | 0.1443 | 0.05 | 5.45E-10 |
| rs17396055 | A | G | -0.1654 | 0.0462 | 3.96E-08 |
| rs76719272 | T | C | -0.1708 | 0.0651 | 2.97E-09 |
| rs1043069 | T | G | 0.1287 | 0.0446 | 5.26E-14 |
| rs4651224 | T | C | 0.186 | 0.0433 | 9E-11 |
| rs12042924 | T | C | -0.1235 | 0.0427 | 2.62E-09 |
| rs33996239 | T | C | -0.3263 | 0.1012 | 3.39E-08 |
| rs7555285 | C | G | 0.1334 | 0.0516 | 1.05E-09 |
| rs4926499 | C | G | 0.2925 | 0.072 | 1.33E-11 |
| rs3802517 | A | T | 0.188 | 0.0456 | 4.65E-17 |
| rs34130368 | T | G | -0.2205 | 0.0647 | 1.28E-09 |
| rs12572586 | T | C | -0.3177 | 0.0874 | 1.23E-09 |
| rs77413490 | T | G | 0.522 | 0.1091 | 4.27E-09 |
| rs11187142 | T | C | 0.298 | 0.0763 | 2.53E-11 |
| rs932764 | A | G | -0.3654 | 0.0467 | 5.43E-45 |
| rs112184198 | A | G | -0.5331 | 0.0761 | 1.94E-40 |
| rs11191548 | T | C | 1.0233 | 0.0818 | 1.16E-87 |
| rs11197813 | A | G | -0.1765 | 0.046 | 3.53E-08 |
| rs11592107 | A | G | 0.2721 | 0.0495 | 1.55E-20 |
| rs72834453 | T | G | -0.2378 | 0.0712 | 2.95E-12 |
| rs1133400 | A | G | -0.193 | 0.051 | 2.53E-15 |
| rs661348 | T | C | -0.3417 | 0.0502 | 5.23E-45 |
| rs10743086 | A | G | -0.14 | 0.0522 | 3.6E-08 |
| rs7129220 | A | G | 0.3919 | 0.0724 | 2.96E-26 |
| rs5219 | T | C | 0.32 | 0.0471 | 9.18E-29 |
| rs10766533 | A | T | 0.2572 | 0.0515 | 4.69E-10 |
| rs871004 | A | G | 0.1329 | 0.0447 | 1.65E-13 |
| rs11031051 | A | C | -0.127 | 0.0445 | 7.73E-12 |
| rs1585453 | A | T | -0.4052 | 0.0761 | 6.15E-15 |
| rs11537751 | T | C | 0.3936 | 0.1076 | 6.3E-11 |
| rs75905900 | A | C | 0.328 | 0.0647 | 1.36E-20 |
| rs11229457 | T | C | -0.2886 | 0.0563 | 8.45E-18 |
| rs4980515 | T | C | 0.1722 | 0.0447 | 9.73E-14 |
| rs3741378 | T | C | -0.4169 | 0.0696 | 4.83E-20 |
| rs67976715 | C | G | 0.1742 | 0.0517 | 6.8E-09 |
| rs7927515 | A | C | 0.1705 | 0.0488 | 1.05E-12 |
| rs4754196 | A | G | -0.1756 | 0.0415 | 1.46E-30 |
| rs1076485 | T | C | 0.2665 | 0.0606 | 1.19E-13 |
| rs78998485 | C | G | -0.1262 | 0.0474 | 1.48E-12 |
| rs2024385 | A | T | -0.1588 | 0.0468 | 5.88E-18 |
| rs28621435 | A | G | -0.3138 | 0.0729 | 6.47E-10 |
| rs7976167 | T | C | 0.1337 | 0.0465 | 3.81E-08 |
| rs1126930 | C | G | 0.5757 | 0.14 | 9.48E-14 |
| rs73099903 | T | C | 0.4218 | 0.0878 | 3.43E-17 |
| rs7297416 | A | C | 0.2816 | 0.05 | 2.9E-30 |
| rs10437954 | A | G | -0.2398 | 0.0697 | 1.6E-14 |
| rs4143175 | T | C | 0.3055 | 0.0533 | 5.1E-10 |
| rs7963801 | T | C | -0.1327 | 0.045 | 2.87E-14 |
| rs17249754 | A | G | -0.8015 | 0.0619 | 1.25E-97 |
| rs10858966 | C | G | 0.248 | 0.0509 | 9.27E-15 |
| rs11112548 | A | T | 0.3673 | 0.1067 | 3.34E-11 |
| rs117206641 | T | C | 0.3348 | 0.0783 | 2.66E-10 |
| rs2480171 | T | C | 0.2057 | 0.0693 | 4.69E-10 |
| rs606950 | A | G | 0.1585 | 0.0434 | 3.23E-18 |
| rs1331012 | T | G | 0.1514 | 0.051 | 1.49E-09 |
| rs63418562 | T | C | -0.3846 | 0.0529 | 6.34E-24 |
| rs9532243 | A | C | 0.3595 | 0.0413 | 8.17E-14 |
| rs4274337 | A | G | -0.33 | 0.0612 | 2.48E-13 |
| rs73187288 | A | C | -0.2105 | 0.0712 | 1.04E-08 |
| rs912434 | T | G | 0.1772 | 0.0497 | 2.47E-11 |
| rs9526707 | A | G | -0.1739 | 0.0456 | 2.77E-10 |
| rs75961402 | A | G | 0.2759 | 0.0635 | 1.95E-10 |
| rs78474310 | A | G | -0.5088 | 0.1041 | 1.51E-10 |
| rs7331680 | T | G | 0.3414 | 0.0583 | 3.35E-22 |
| rs17115145 | T | C | 0.1159 | 0.0433 | 7.39E-09 |
| rs34983854 | A | G | -0.2259 | 0.0463 | 2.06E-11 |
| rs72683923 | T | C | 1.0239 | 0.1767 | 3.08E-18 |
| rs9888615 | T | C | -0.2356 | 0.0499 | 1.46E-16 |
| rs11623535 | A | G | 0.1885 | 0.0471 | 1.02E-09 |
| rs11159091 | A | G | 0.1433 | 0.0422 | 6.79E-11 |
| rs8014182 | T | C | -0.3509 | 0.062 | 5.23E-14 |
| rs11629850 | A | G | 0.1417 | 0.0416 | 2.26E-14 |
| rs2759308 | A | G | 0.2592 | 0.046 | 1.89E-25 |
| rs3743157 | A | C | 0.1997 | 0.0549 | 4.2E-13 |
| rs11632436 | C | G | 0.1738 | 0.042 | 1.96E-13 |
| rs2379829 | C | G | -0.2143 | 0.0521 | 4.48E-15 |
| rs34941092 | A | G | -0.302 | 0.0651 | 3.23E-14 |
| rs1012089 | C | G | -0.1354 | 0.0456 | 1.95E-10 |
| rs7187540 | A | C | -0.1431 | 0.0458 | 1.02E-08 |
| rs3851018 | C | G | 0.2224 | 0.0473 | 5.4E-10 |
| rs6540125 | T | G | 0.1864 | 0.0475 | 1.21E-10 |
| rs4480845 | T | C | 0.1473 | 0.0447 | 1.85E-23 |
| rs4925159 | A | G | 0.2482 | 0.0419 | 9.66E-13 |
| rs1551355 | T | C | 0.1618 | 0.049 | 3.89E-09 |
| rs9899540 | A | T | 0.1809 | 0.0487 | 1.87E-10 |
| rs12946454 | A | T | -0.3193 | 0.0518 | 1.01E-33 |
| rs7406910 | T | C | -0.4877 | 0.0812 | 3.39E-16 |
| rs34430710 | A | T | -0.1972 | 0.0459 | 5.02E-11 |
| rs1036902 | T | C | -0.1786 | 0.0566 | 1.7E-09 |
| rs2240736 | T | C | 0.4265 | 0.0525 | 1.17E-32 |
| rs6504213 | T | C | -0.1381 | 0.0437 | 1.25E-21 |
| rs112260610 | T | C | 0.3389 | 0.0669 | 2.69E-09 |
| rs9302885 | A | G | 0.1367 | 0.0421 | 1.03E-13 |
| rs112280096 | A | C | -0.1169 | 0.0431 | 1.33E-09 |
| rs34413141 | A | T | -0.2124 | 0.0576 | 2.47E-19 |
| rs1154214 | T | G | -0.2163 | 0.046 | 3.27E-11 |
| rs12958173 | A | C | 0.3518 | 0.0495 | 2.97E-19 |
| rs11876341 | A | G | -0.2406 | 0.0461 | 1.82E-10 |
| rs10048404 | T | C | -0.1412 | 0.0513 | 1.91E-16 |
| rs6567160 | T | C | 0.1618 | 0.0541 | 3.33E-10 |
| rs12454712 | T | C | 0.2258 | 0.0426 | 5.82E-09 |
| rs10460108 | A | G | 0.2039 | 0.0452 | 1.12E-12 |
| rs2613765 | A | G | -0.2304 | 0.0415 | 5.32E-15 |
| rs4247374 | T | C | -0.5063 | 0.0753 | 4.52E-44 |
| rs10409243 | T | C | -0.1088 | 0.0443 | 8.1E-23 |
| rs17638167 | T | C | -0.5228 | 0.1095 | 1.07E-09 |
| rs8105753 | A | C | 0.1895 | 0.0487 | 6.84E-12 |
| rs7256564 | A | G | 0.2039 | 0.0487 | 1.53E-09 |
| rs73046792 | A | G | -0.2413 | 0.069 | 7.23E-17 |
| rs67720684 | A | C | 0.2242 | 0.0512 | 3.85E-08 |
| rs1344653 | A | G | -0.1568 | 0.0456 | 3.09E-17 |
| rs55701159 | T | G | 0.2999 | 0.0742 | 1.73E-16 |
| rs1275988 | T | C | -0.5157 | 0.0466 | 4.42E-69 |
| rs9678851 | A | C | -0.1135 | 0.0474 | 1.99E-08 |
| rs7562 | T | C | 0.1555 | 0.047 | 3.26E-14 |
| rs13420463 | A | G | 0.2751 | 0.0555 | 2.72E-18 |
| rs35590893 | A | G | -0.1706 | 0.0477 | 1.66E-12 |
| rs10189186 | A | G | 0.1752 | 0.0459 | 3.91E-10 |
| rs72816333 | A | T | 0.2016 | 0.0549 | 5.45E-09 |
| rs2300481 | T | C | 0.1887 | 0.0427 | 1.56E-10 |
| rs72847885 | A | G | 0.1717 | 0.0452 | 3.08E-14 |
| rs28377357 | A | G | -0.1335 | 0.0454 | 9.6E-11 |
| rs6723509 | T | C | 0.2018 | 0.0588 | 7.61E-09 |
| rs55732192 | T | G | -0.2807 | 0.0798 | 1.15E-10 |
| rs1446468 | T | C | -0.487 | 0.0468 | 4.38E-62 |
| rs6712203 | T | C | -0.1943 | 0.0477 | 2.41E-11 |
| rs11694601 | A | G | -0.1422 | 0.047 | 6.41E-10 |
| rs1837164 | A | T | 0.186 | 0.0472 | 4.66E-09 |
| rs28558491 | T | C | -0.1419 | 0.0473 | 7.54E-10 |
| rs296797 | T | C | 0.2067 | 0.0467 | 2.16E-12 |
| rs55780018 | T | C | -0.3278 | 0.0488 | 2.73E-19 |
| rs1047891 | A | C | -0.1647 | 0.0511 | 1.37E-14 |
| rs12694277 | T | C | -0.1284 | 0.0462 | 1.8E-09 |
| rs2972146 | T | G | 0.2486 | 0.0476 | 6.53E-16 |
| rs1044822 | T | C | -0.1852 | 0.0608 | 5.16E-09 |
| rs139354822 | T | C | 0.442 | 0.1248 | 3.51E-10 |
| rs1764975 | A | T | 0.2759 | 0.058 | 1.08E-13 |
| rs6031435 | A | G | -0.2268 | 0.0456 | 1.09E-17 |
| rs6021247 | A | G | 0.1506 | 0.0411 | 9.8E-15 |
| rs1882961 | T | C | 0.2147 | 0.0466 | 6.69E-14 |
| rs11701033 | C | G | -0.2465 | 0.0592 | 2.52E-08 |
| rs9608690 | A | G | -0.308 | 0.0912 | 6.13E-10 |
| rs28578714 | T | C | 0.1761 | 0.0421 | 2.53E-10 |
| rs347591 | T | G | 0.2842 | 0.0489 | 2.87E-23 |
| rs11128722 | A | G | -0.2518 | 0.047 | 1.93E-20 |
| rs189267552 | A | T | -0.5985 | 0.2068 | 4.55E-10 |
| rs12638085 | A | T | 0.2004 | 0.0479 | 5.61E-12 |
| rs6788984 | A | G | 0.168 | 0.061 | 3.81E-12 |
| rs4499560 | A | T | -0.1637 | 0.0457 | 1.46E-11 |
| rs9857362 | A | C | 0.1104 | 0.0418 | 1.62E-08 |
| rs1375564 | T | C | 0.1884 | 0.0439 | 2.84E-16 |
| rs6438857 | T | C | 0.0977 | 0.0427 | 3.13E-19 |
| rs9875380 | T | C | -0.1456 | 0.0421 | 6.53E-09 |
| rs143112823 | A | G | -0.4019 | 0.0949 | 7.16E-14 |
| rs78151625 | T | C | -0.1451 | 0.0562 | 1.61E-09 |
| rs262986 | A | G | -0.2288 | 0.0468 | 7.67E-15 |
| rs231708 | C | G | -0.1252 | 0.0449 | 4.74E-18 |
| rs2610990 | A | G | -0.1915 | 0.0484 | 2.86E-17 |
| rs2291435 | T | C | -0.2419 | 0.0463 | 5.31E-18 |
| rs12511987 | T | G | -0.1817 | 0.0551 | 5.39E-09 |
| rs10008637 | T | C | 0.1355 | 0.0415 | 9.24E-13 |
| rs2014912 | T | C | 0.5122 | 0.0644 | 6.97E-30 |
| rs13149209 | T | C | 0.196 | 0.0496 | 1.97E-14 |
| rs1347345 | A | G | -0.1765 | 0.0433 | 6.92E-09 |
| rs13112725 | C | G | 0.397 | 0.0557 | 6.81E-31 |
| rs3097937 | A | T | 0.2388 | 0.0587 | 4.95E-09 |
| rs7439567 | T | C | 0.1998 | 0.0432 | 2.31E-16 |
| rs72719160 | A | T | -0.2509 | 0.0444 | 4.34E-12 |
| rs6823767 | T | C | -0.1566 | 0.0528 | 4.4E-10 |
| rs17035181 | T | G | 0.271 | 0.0593 | 7.61E-13 |
| rs4957026 | A | G | 0.2214 | 0.0497 | 8.12E-10 |
| rs10069690 | T | C | 0.2151 | 0.0468 | 4.47E-17 |
| rs1173771 | A | G | -0.5227 | 0.0468 | 5.92E-94 |
| rs1694068 | A | T | 0.1739 | 0.0427 | 1.18E-17 |
| rs13179413 | T | C | 0.1656 | 0.0492 | 1.08E-10 |
| rs6875372 | A | T | 0.2228 | 0.0459 | 4.8E-10 |
| rs246973 | T | C | 0.1721 | 0.0499 | 1.45E-13 |
| rs10059921 | T | G | -0.3732 | 0.0919 | 4.39E-13 |
| rs709668 | A | G | -0.1743 | 0.0524 | 5.96E-15 |
| rs1871190 | T | G | 0.1658 | 0.0495 | 1.66E-09 |
| rs10077885 | A | C | -0.2465 | 0.0484 | 4.43E-17 |
| rs13359291 | A | G | 0.4005 | 0.062 | 1.64E-25 |
| rs62373688 | A | T | 0.2301 | 0.0684 | 1.58E-09 |
| rs6595838 | A | G | 0.2361 | 0.0507 | 1.54E-22 |
| rs702395 | T | C | 0.115 | 0.0416 | 3.24E-14 |
| rs1650911 | C | G | 0.2465 | 0.0584 | 4.34E-12 |
| rs11953630 | T | C | -0.4463 | 0.0501 | 1.55E-45 |
| rs12153395 | A | G | -0.2602 | 0.0764 | 1.07E-11 |
| rs2745599 | A | G | 0.1464 | 0.0418 | 8.96E-12 |
| rs9368222 | A | C | 0.2337 | 0.0462 | 1.84E-11 |
| rs6911827 | T | C | 0.152 | 0.0473 | 7.96E-15 |
| rs1563788 | T | C | 0.3062 | 0.0501 | 2.36E-24 |
| rs78648104 | T | C | -0.3571 | 0.083 | 2.37E-15 |
| rs9449350 | T | C | -0.2333 | 0.0488 | 1.19E-11 |
| rs35410524 | T | C | 0.2999 | 0.0588 | 3.19E-18 |
| rs10782230 | A | G | 0.1776 | 0.0425 | 2.91E-12 |
| rs9885632 | T | C | 0.1582 | 0.0486 | 4.37E-12 |
| rs7763294 | T | G | -0.1418 | 0.045 | 6.39E-10 |
| rs7765526 | A | G | 0.2317 | 0.047 | 5.88E-11 |
| rs6959688 | A | G | -0.1323 | 0.0437 | 4.22E-14 |
| rs12979 | C | G | 0.2241 | 0.0693 | 1.09E-09 |
| rs6969780 | C | G | 0.3697 | 0.0793 | 1.88E-08 |
| rs6963105 | A | G | -0.1548 | 0.046 | 3.83E-09 |
| rs848445 | T | C | -0.196 | 0.049 | 2.28E-09 |
| rs17477177 | T | C | -0.5642 | 0.0564 | 9.01E-86 |
| rs4728142 | A | G | -0.2155 | 0.0467 | 2.59E-09 |
| rs34072724 | A | G | -0.1735 | 0.0412 | 1.37E-15 |
| rs13238550 | A | G | 0.1695 | 0.0472 | 7.8E-17 |
| rs11771693 | A | G | 0.1368 | 0.0455 | 1.9E-08 |
| rs10224002 | A | G | -0.2375 | 0.0525 | 1.31E-27 |
| rs1870735 | C | G | 0.2137 | 0.0486 | 3.61E-11 |
| rs4875958 | A | G | 0.2209 | 0.0515 | 1.85E-11 |
| rs61040371 | T | C | 0.191 | 0.0475 | 4.77E-09 |
| rs62491354 | A | G | 0.1582 | 0.0608 | 3.25E-12 |
| rs1986971 | A | G | 0.1991 | 0.0478 | 1.61E-14 |
| rs2898290 | T | C | 0.3419 | 0.0466 | 1.08E-24 |
| rs6557876 | T | C | -0.3667 | 0.0533 | 1.11E-32 |
| rs2979470 | T | C | 0.2114 | 0.046 | 4.62E-11 |
| rs1906672 | A | G | 0.2227 | 0.0499 | 1.2E-16 |
| rs4873492 | T | C | 0.2131 | 0.0584 | 1.61E-17 |
| rs2354862 | A | C | 0.2139 | 0.0485 | 2.42E-15 |
| rs13253358 | T | C | 0.1945 | 0.0504 | 1.13E-10 |
| rs72688070 | T | C | -0.187 | 0.0549 | 2.82E-11 |
| rs62526122 | A | G | 0.1739 | 0.0557 | 1.02E-09 |
| rs142449193 | T | C | -0.4354 | 0.112 | 7.86E-10 |
| rs35783704 | A | G | -0.5219 | 0.0773 | 8.81E-20 |
| rs62523863 | A | G | 0.1643 | 0.0502 | 2.87E-13 |
| rs4598218 | T | C | 0.1578 | 0.0435 | 1E-09 |
| rs4129585 | A | C | 0.1541 | 0.0414 | 1.03E-09 |
| rs520015 | C | G | 0.2043 | 0.0456 | 2.84E-11 |
| rs60191654 | A | G | -0.2311 | 0.0584 | 5.88E-10 |
| rs28558845 | C | G | -0.1476 | 0.0568 | 1.16E-09 |
| rs1332813 | T | C | 0.1453 | 0.046 | 2.32E-12 |
| rs9886665 | T | C | 0.1887 | 0.0519 | 2.47E-09 |
| rs7045409 | A | T | -0.1746 | 0.0441 | 2.55E-09 |
| rs111245230 | T | C | -0.6917 | 0.1299 | 2.81E-19 |
| rs7023828 | T | C | -0.2125 | 0.0427 | 2.17E-17 |
| rs1891730 | T | C | -0.1733 | 0.0441 | 7.74E-09 |

EA, effect allele; NEA, non-effect allele

*P value taken from the final meta-analysis

**Supplementary Table S4. Genetic variants for fasting glucose**

| **rsID** | **EA** | **NEA** | **Effect size** | **Standard error** | **P** |
| --- | --- | --- | --- | --- | --- |
| rs780093 | T | C | -0.032 | 0.0031 | 4.23E-25 |
| rs477309 | T | C | 0.024 | 0.0042 | 2.54E-08 |
| rs560887 | T | C | -0.071 | 0.0034 | 2.6E-99 |
| rs12053049 | T | C | -0.096 | 0.015 | 3.17E-10 |
| rs16843390 | A | G | 0.23 | 0.041 | 3.32E-08 |
| rs11708067 | A | G | 0.022 | 0.0038 | 6.79E-09 |
| rs7644261 | C | G | 0.021 | 0.0034 | 8.18E-10 |
| rs4869272 | T | C | 0.02 | 0.0033 | 2.7E-09 |
| rs10276674 | T | C | -0.039 | 0.0043 | 7.77E-20 |
| rs2191349 | T | G | 0.028 | 0.0031 | 1.98E-20 |
| rs882020 | T | C | 0.029 | 0.0045 | 1.36E-10 |
| rs2908282 | A | G | 0.064 | 0.0042 | 3.51E-53 |
| rs983309 | T | G | 0.03 | 0.0048 | 7.61E-10 |
| rs11558471 | A | G | 0.029 | 0.0034 | 7.07E-18 |
| rs10974438 | A | C | -0.019 | 0.0033 | 5.23E-09 |
| rs11195502 | T | C | -0.035 | 0.0054 | 1.04E-10 |
| rs17747324 | T | C | -0.024 | 0.0039 | 7.5E-10 |
| rs11605924 | A | C | 0.022 | 0.0031 | 2.99E-13 |
| rs7944584 | A | T | 0.025 | 0.0035 | 4.38E-13 |
| rs1483121 | A | G | -0.029 | 0.0048 | 1.66E-09 |
| rs2524299 | A | T | 0.029 | 0.0044 | 2.59E-11 |
| rs11603334 | A | G | -0.023 | 0.0041 | 2.92E-08 |
| rs10830963 | C | G | -0.079 | 0.0037 | 2.2E-100 |
| rs7173964 | A | G | -0.02 | 0.0031 | 1.04E-10 |
| rs7172969 | A | C | -0.81 | 0.14 | 4.2E-09 |
| rs6113722 | A | G | -0.042 | 0.0077 | 3.89E-08 |

EA, effect allele; NEA, non-effect allele

**Supplementary Table S5. Genetic variants for HbA1c**

| **rsID** | **EA** | **NEA** | **Effect size** | **Standard error** | **P** |
| --- | --- | --- | --- | --- | --- |
| rs267738 | G | T | -0.011 | 0.0019 | 2.59E-09 |
| rs2246434 | A | G | 0.019 | 0.0018 | 1.99E-27 |
| rs5030913 | G | T | -0.013 | 0.0018 | 3.56E-13 |
| rs4745982 | G | T | -0.095 | 0.0056 | 2.87E-65 |
| rs17747324 | C | T | 0.015 | 0.0023 | 6.12E-11 |
| rs3782123 | A | C | -0.013 | 0.002 | 1.51E-10 |
| rs11603334 | A | G | -0.012 | 0.0021 | 6.85E-09 |
| rs1387153 | C | T | -0.019 | 0.0019 | 2.11E-24 |
| rs2110073 | C | T | -0.015 | 0.0028 | 4.44E-08 |
| rs2408955 | G | T | -0.012 | 0.0016 | 1.42E-15 |
| rs10774625 | A | G | -0.0088 | 0.0016 | 1.46E-08 |
| rs17630235 | A | G | -0.0086 | 0.0016 | 3.08E-08 |
| rs423117 | C | T | -0.019 | 0.0027 | 1.3E-12 |
| rs11248914 | C | T | -0.014 | 0.0019 | 2.56E-14 |
| rs9935401 | A | G | 0.0099 | 0.0018 | 1.87E-08 |
| rs837763 | C | T | -0.017 | 0.0016 | 1.68E-28 |
| rs9914988 | A | G | 0.013 | 0.002 | 2.77E-11 |
| rs1046896 | C | T | -0.028 | 0.0017 | 4.46E-64 |
| rs17533903 | A | G | 0.015 | 0.0022 | 5.27E-12 |
| rs17509001 | C | T | 0.018 | 0.0023 | 1.94E-15 |
| rs12621844 | C | T | -0.0099 | 0.0018 | 1.87E-08 |
| rs560887 | C | T | 0.028 | 0.0018 | 1.48E-58 |
| rs855791 | A | G | 0.017 | 0.0016 | 3.44E-28 |
| rs7616006 | A | G | 0.01 | 0.0017 | 5.07E-10 |
| rs9818758 | A | G | 0.012 | 0.002 | 7.74E-10 |
| rs11708067 | A | G | 0.013 | 0.0019 | 1.42E-12 |
| rs8192675 | C | T | -0.011 | 0.0017 | 1.38E-11 |
| rs13134327 | A | G | 0.013 | 0.0017 | 2.64E-15 |
| rs7756992 | A | G | -0.012 | 0.0018 | 2.8E-12 |
| rs17492120 | C | T | 0.017 | 0.0026 | 5.13E-11 |
| rs1800562 | A | G | -0.04 | 0.0036 | 4.67E-28 |
| rs13194491 | C | T | 0.021 | 0.0034 | 1.15E-09 |
| rs13217599 | C | T | -0.021 | 0.0035 | 4.11E-09 |
| rs11964178 | A | G | 0.0096 | 0.0016 | 6.38E-10 |
| rs9494142 | C | T | -0.021 | 0.0024 | 7.45E-18 |
| rs592423 | A | C | 0.0091 | 0.0017 | 3.96E-08 |
| rs2979422 | C | T | 0.015 | 0.0023 | 1.1E-10 |
| rs4607517 | A | G | 0.031 | 0.0024 | 8.76E-38 |
| rs6474359 | C | T | -0.044 | 0.0053 | 1.5E-16 |
| rs4737009 | A | G | 0.021 | 0.002 | 4.48E-27 |
| rs6980507 | A | G | 0.0097 | 0.0018 | 3.58E-08 |
| rs13266634 | C | T | 0.015 | 0.0017 | 4.53E-20 |
| rs2383208 | A | G | 0.014 | 0.0021 | 7.04E-12 |
| rs7040409 | C | G | 0.028 | 0.0037 | 2.56E-14 |
| rs579459 | C | T | 0.011 | 0.0019 | 9.42E-09 |

HbA1c, glycated haemoglobin; EA, effect allele; NEA, non-effect allele

**Supplementary Table S6. Genetic variants for LDL cholesterol**

| **rsID** | **EA** | **NEA** | **Effect size** | **Standard error** | **P** |
| --- | --- | --- | --- | --- | --- |
| rs2419604 | A | G | 0.0302 | 0.004 | 7.49E-14 |
| rs413380 | C | T | 0.0861 | 0.0098 | 7.62E-17 |
| rs4970834 | C | T | 0.1503 | 0.0047 | 1E-200 |
| rs9804646 | C | T | 0.0454 | 0.007 | 8.6E-11 |
| rs10893499 | A | G | 0.0521 | 0.0053 | 3.86E-21 |
| rs10832962 | T | C | 0.032 | 0.004 | 6.62E-14 |
| rs267733 | A | G | 0.0331 | 0.0053 | 5.29E-09 |
| rs174583 | C | T | 0.0522 | 0.0038 | 7E-41 |
| rs3184504 | C | T | 0.0268 | 0.0038 | 4.2E-12 |
| rs1169288 | C | A | 0.0375 | 0.004 | 6.45E-21 |
| rs2642438 | G | A | 0.0352 | 0.0042 | 7.32E-16 |
| rs2587534 | A | G | 0.0391 | 0.0037 | 8.06E-25 |
| rs10903129 | G | A | 0.0328 | 0.0037 | 3.03E-17 |
| rs12748152 | T | C | 0.0499 | 0.0066 | 3.21E-12 |
| rs4942486 | T | C | 0.0243 | 0.0037 | 2.26E-11 |
| rs8017377 | A | G | 0.0303 | 0.0038 | 2.52E-15 |
| rs11206508 | A | G | 0.0434 | 0.0055 | 2.26E-14 |
| rs17111503 | G | A | 0.0662 | 0.0045 | 1.39E-45 |
| rs11591147 | G | T | 0.497 | 0.018 | 8.6E-143 |
| rs630431 | A | G | 0.0351 | 0.0042 | 7.73E-17 |
| rs11583974 | A | G | 0.0646 | 0.0117 | 3.95E-09 |
| rs2647281 | G | A | 0.0589 | 0.0095 | 2.27E-09 |
| rs207150 | C | T | 0.0472 | 0.0065 | 2E-12 |
| rs11485618 | A | G | 0.05 | 0.0039 | 3.73E-33 |
| rs247616 | C | T | 0.0547 | 0.0041 | 2.57E-37 |
| rs2000999 | A | G | 0.065 | 0.0046 | 4.22E-41 |
| rs6504872 | T | C | 0.0274 | 0.0037 | 3.48E-13 |
| rs1801689 | C | A | 0.1028 | 0.0139 | 9.81E-12 |
| rs2886232 | T | C | 0.0451 | 0.0064 | 3.88E-11 |
| rs314253 | T | C | 0.0242 | 0.0038 | 3.44E-10 |
| rs11669133 | A | G | 0.0501 | 0.0098 | 4.8E-08 |
| rs6511720 | G | T | 0.2209 | 0.0061 | 1E-200 |
| rs688 | T | C | 0.054 | 0.0037 | 1.01E-43 |
| rs6511727 | T | G | 0.0266 | 0.0038 | 1.84E-11 |
| rs376642 | C | T | 0.0233 | 0.004 | 4.67E-10 |
| rs10401969 | T | C | 0.1184 | 0.0072 | 2.65E-54 |
| rs4970712 | C | A | 0.0339 | 0.0044 | 2.46E-13 |
| rs17800760 | G | A | 0.0513 | 0.0053 | 8.87E-22 |
| rs10460181 | A | G | 0.0536 | 0.0046 | 2.25E-28 |
| rs1531517 | G | A | 0.2202 | 0.008 | 9.5E-163 |
| rs7254892 | G | A | 0.4853 | 0.0119 | 1E-200 |
| rs2075650 | G | A | 0.1767 | 0.0055 | 1E-200 |
| rs75687619 | T | G | 0.1735 | 0.0161 | 8.05E-24 |
| rs2287019 | C | T | 0.0283 | 0.0048 | 8.36E-09 |
| rs492602 | G | A | 0.0293 | 0.0039 | 9.42E-14 |
| rs364585 | G | A | 0.0249 | 0.0038 | 4.28E-10 |
| rs2328223 | C | A | 0.0299 | 0.005 | 5.63E-09 |
| rs7264396 | C | T | 0.0246 | 0.0045 | 4.41E-08 |
| rs6016381 | T | C | 0.0363 | 0.0038 | 6.85E-20 |
| rs6065311 | C | T | 0.0417 | 0.0036 | 1.66E-30 |
| rs1800961 | C | T | 0.0685 | 0.0106 | 6.03E-10 |
| rs10490626 | G | A | 0.0508 | 0.0069 | 1.7E-12 |
| rs2030746 | T | C | 0.0214 | 0.0038 | 8.61E-09 |
| rs16831243 | T | C | 0.0378 | 0.0055 | 9.06E-12 |
| rs10195252 | T | C | 0.0238 | 0.0039 | 3.81E-08 |
| rs492399 | G | A | 0.0629 | 0.0102 | 1.23E-09 |
| rs13414987 | A | C | 0.0308 | 0.0043 | 9.94E-12 |
| rs1367117 | A | G | 0.1186 | 0.004 | 9.5E-183 |
| rs12471982 | C | A | 0.0365 | 0.0054 | 3.93E-11 |
| rs520861 | G | A | 0.0843 | 0.0042 | 1.78E-84 |
| rs1250229 | C | T | 0.0243 | 0.0042 | 3.13E-08 |
| rs5763662 | T | C | 0.0767 | 0.0121 | 1.19E-08 |
| rs11563251 | T | C | 0.0345 | 0.0062 | 4.5E-08 |
| rs4253776 | G | A | 0.0311 | 0.0059 | 3.35E-08 |
| rs780093 | T | C | 0.0223 | 0.0037 | 2.36E-08 |
| rs1025447 | C | T | 0.0418 | 0.0048 | 3.78E-16 |
| rs6544713 | T | C | 0.0806 | 0.0041 | 4.84E-83 |
| rs6709904 | A | G | 0.055 | 0.0085 | 4.58E-10 |
| rs2710642 | A | G | 0.0239 | 0.0038 | 6.09E-09 |
| rs9875338 | G | A | 0.027 | 0.0037 | 2.21E-11 |
| rs17404153 | G | T | 0.0336 | 0.0054 | 1.83E-09 |
| rs7640978 | C | T | 0.0392 | 0.0069 | 9.84E-09 |
| rs6818397 | T | G | 0.0224 | 0.004 | 1.68E-08 |
| rs4530754 | A | G | 0.0275 | 0.0036 | 3.58E-12 |
| rs6882076 | C | T | 0.0456 | 0.0038 | 3.31E-31 |
| rs12916 | C | T | 0.0733 | 0.0038 | 7.79E-78 |
| rs6909746 | C | T | 0.0263 | 0.0037 | 7.86E-11 |
| rs1564348 | C | T | 0.0481 | 0.005 | 2.76E-21 |
| rs3125055 | A | T | 0.0468 | 0.0055 | 5.92E-16 |
| rs1510226 | C | T | 0.1409 | 0.0214 | 1.71E-10 |
| rs7770628 | C | T | 0.0258 | 0.0037 | 3.17E-11 |
| rs3757354 | C | T | 0.0382 | 0.0044 | 2.09E-17 |
| rs13206249 | G | A | 0.0378 | 0.0062 | 4.53E-08 |
| rs1800562 | G | A | 0.0615 | 0.008 | 8.25E-14 |
| rs2247056 | C | T | 0.0248 | 0.0043 | 1.42E-08 |
| rs10947332 | A | G | 0.0504 | 0.0056 | 6.97E-18 |
| rs12670798 | C | T | 0.0344 | 0.0043 | 4.81E-14 |
| rs4722551 | C | T | 0.0391 | 0.0049 | 3.95E-14 |
| rs2073547 | G | A | 0.0485 | 0.0049 | 1.92E-21 |
| rs2737252 | G | A | 0.0314 | 0.0041 | 7.04E-14 |
| rs2954029 | A | T | 0.0564 | 0.0036 | 2.1E-50 |
| rs7832643 | T | G | 0.0339 | 0.0038 | 2.67E-17 |
| rs10102164 | A | G | 0.0316 | 0.0045 | 3.74E-11 |
| rs13277801 | C | T | 0.0338 | 0.0038 | 3.99E-17 |
| rs9987289 | G | A | 0.0714 | 0.0066 | 8.53E-24 |
| rs1883025 | C | T | 0.0296 | 0.0044 | 6.14E-11 |
| rs8176722 | C | A | 0.0473 | 0.006 | 1.85E-14 |
| rs579459 | C | T | 0.0665 | 0.0045 | 2.42E-44 |
| rs3780181 | A | G | 0.0445 | 0.0074 | 1.76E-09 |
| rs519113 | C | G | 0.0971 | 0.0066 | 1.61E-49 |
| rs964184 | G | C | 0.0855 | 0.0078 | 2.01E-26 |

LDL, low-density lipoprotein; EA, effect allele; NEA, non-effect allele

**Supplementary Table S7. Genetic variants for HDL cholesterol**

| **rsID** | **EA** | **NEA** | **Effect size** | **Standard error** | **P** |
| --- | --- | --- | --- | --- | --- |
| rs2250802 | G | A | 0.034 | 0.0038 | 2.02E-17 |
| rs2148489 | T | C | 0.0283 | 0.0041 | 1.41E-10 |
| rs970548 | C | A | 0.0258 | 0.0039 | 1.71E-10 |
| rs10761771 | C | T | 0.0198 | 0.0034 | 4.12E-09 |
| rs12740374 | T | G | 0.0343 | 0.0041 | 1.69E-15 |
| rs333947 | G | A | 0.0296 | 0.0047 | 3.17E-09 |
| rs7943309 | A | G | 0.0865 | 0.0088 | 1.18E-20 |
| rs7117842 | C | T | 0.0272 | 0.0035 | 1.06E-14 |
| rs17135399 | A | G | 0.0483 | 0.0077 | 4.26E-09 |
| rs7128597 | C | A | 0.0398 | 0.0065 | 1.82E-08 |
| rs3847502 | A | C | 0.048 | 0.0036 | 3.31E-38 |
| rs4752894 | G | A | 0.0206 | 0.0035 | 1.89E-09 |
| rs12145743 | G | T | 0.0203 | 0.0036 | 1.8E-08 |
| rs102275 | T | C | 0.0391 | 0.0035 | 6.4E-28 |
| rs12801636 | A | G | 0.0235 | 0.0042 | 3.15E-08 |
| rs499974 | C | A | 0.0263 | 0.0044 | 1.12E-08 |
| rs4650994 | G | A | 0.021 | 0.0034 | 6.7E-09 |
| rs1689797 | C | A | 0.0358 | 0.0036 | 2.85E-21 |
| rs2241210 | G | A | 0.0332 | 0.0035 | 2.49E-20 |
| rs653178 | T | C | 0.0263 | 0.0035 | 1.06E-12 |
| rs2454722 | G | A | 0.0351 | 0.0044 | 3.31E-14 |
| rs11057397 | T | C | 0.0282 | 0.0036 | 6.77E-14 |
| rs863750 | C | T | 0.0264 | 0.0035 | 4.71E-13 |
| rs838876 | A | G | 0.0493 | 0.0039 | 7.33E-33 |
| rs7306660 | G | A | 0.0345 | 0.0036 | 3.34E-19 |
| rs7298751 | G | A | 0.0434 | 0.0052 | 2.46E-16 |
| rs2642438 | G | A | 0.0303 | 0.0039 | 7.78E-14 |
| rs11045163 | G | A | 0.0217 | 0.0035 | 3.2E-09 |
| rs4846914 | A | G | 0.0479 | 0.0034 | 3.51E-41 |
| rs3741414 | T | C | 0.0296 | 0.004 | 6.1E-14 |
| rs12748152 | C | T | 0.0506 | 0.0062 | 9.74E-16 |
| rs4660293 | A | G | 0.0353 | 0.004 | 2.86E-18 |
| rs4983559 | G | A | 0.0197 | 0.0036 | 9.57E-09 |
| rs492571 | T | C | 0.0663 | 0.009 | 1.27E-12 |
| rs2899624 | A | G | 0.0714 | 0.0049 | 1.39E-40 |
| rs185481 | C | T | 0.0366 | 0.0035 | 1.4E-23 |
| rs16940147 | A | G | 0.0514 | 0.008 | 2.45E-10 |
| rs10468017 | T | C | 0.1179 | 0.0038 | 1.2E-188 |
| rs1077834 | C | T | 0.1253 | 0.0041 | 7.8E-180 |
| rs424346 | T | C | 0.0679 | 0.0113 | 4.84E-08 |
| rs1007076 | C | T | 0.0247 | 0.0041 | 4.43E-09 |
| rs1121980 | G | A | 0.0196 | 0.0034 | 6.79E-09 |
| rs3790106 | C | G | 0.0374 | 0.0052 | 3.27E-11 |
| rs4784659 | T | C | 0.0274 | 0.0049 | 1.02E-08 |
| rs13336936 | T | C | 0.0717 | 0.0104 | 6.98E-11 |
| rs7193072 | A | G | 0.0498 | 0.0038 | 1.4E-34 |
| rs1138429 | A | T | 0.1156 | 0.0065 | 9.52E-66 |
| rs9989419 | G | A | 0.1473 | 0.0036 | 1E-200 |
| rs4783961 | A | G | 0.0997 | 0.0036 | 5.7E-162 |
| rs289745 | A | C | 0.0276 | 0.0041 | 2.28E-20 |
| rs291040 | T | C | 0.0305 | 0.0037 | 8.21E-17 |
| rs16942887 | A | G | 0.0831 | 0.0051 | 8.28E-54 |
| rs4986970 | A | T | 0.0792 | 0.0099 | 1.09E-15 |
| rs2925979 | C | T | 0.0351 | 0.0037 | 1.32E-19 |
| rs1877031 | A | G | 0.0336 | 0.0036 | 1.2E-19 |
| rs4148005 | T | G | 0.0283 | 0.0036 | 5.74E-14 |
| rs4969178 | G | A | 0.0263 | 0.0035 | 1.53E-12 |
| rs8093249 | A | G | 0.0384 | 0.0051 | 1.8E-13 |
| rs9955201 | A | G | 0.0638 | 0.0081 | 2.4E-14 |
| rs4939883 | C | T | 0.0799 | 0.0045 | 1.8E-66 |
| rs9951669 | G | A | 0.0408 | 0.0042 | 3.01E-21 |
| rs6567160 | T | C | 0.0257 | 0.0041 | 2.92E-09 |
| rs737337 | T | C | 0.0565 | 0.0061 | 4.56E-17 |
| rs12133576 | A | G | 0.0243 | 0.0035 | 6.15E-11 |
| rs731839 | A | G | 0.022 | 0.0037 | 3.44E-09 |
| rs2075650 | A | G | 0.0554 | 0.0051 | 9.72E-26 |
| rs77301115 | G | A | 0.0972 | 0.0157 | 1.03E-08 |
| rs7412 | T | C | 0.0978 | 0.0097 | 4.44E-19 |
| rs5167 | G | T | 0.032 | 0.0037 | 4.88E-16 |
| rs17695224 | G | A | 0.029 | 0.0039 | 2.42E-13 |
| rs103294 | T | C | 0.0523 | 0.0044 | 4E-30 |
| rs2278236 | A | G | 0.0331 | 0.0035 | 3.19E-18 |
| rs3111576 | T | C | 0.0448 | 0.0054 | 1.2E-14 |
| rs1800961 | C | T | 0.127 | 0.0099 | 1.64E-34 |
| rs4465830 | A | G | 0.0597 | 0.0044 | 5.18E-40 |
| rs17380117 | A | G | 0.0253 | 0.0042 | 3.85E-09 |
| rs7607980 | C | T | 0.0447 | 0.0052 | 1.81E-15 |
| rs676210 | A | G | 0.066 | 0.004 | 2.35E-54 |
| rs1047891 | C | A | 0.0269 | 0.0039 | 8.73E-10 |
| rs181360 | T | G | 0.0376 | 0.0042 | 9.24E-18 |
| rs1515110 | G | T | 0.0323 | 0.0035 | 8.04E-18 |
| rs2606736 | C | T | 0.0246 | 0.0043 | 4.8E-08 |
| rs6805251 | T | C | 0.02 | 0.0035 | 1.33E-08 |
| rs13076253 | A | C | 0.0283 | 0.0048 | 4.96E-09 |
| rs687339 | C | T | 0.0316 | 0.0042 | 7.11E-13 |
| rs2290547 | G | A | 0.0297 | 0.0046 | 3.69E-09 |
| rs2013208 | T | C | 0.0254 | 0.0036 | 8.92E-12 |
| rs13326165 | A | G | 0.0289 | 0.0043 | 9.04E-11 |
| rs2602836 | A | G | 0.0192 | 0.0034 | 4.96E-08 |
| rs13107325 | C | T | 0.0708 | 0.0078 | 1.07E-15 |
| rs10019888 | A | G | 0.027 | 0.0046 | 4.9E-08 |
| rs442177 | G | T | 0.0215 | 0.0034 | 2.19E-09 |
| rs3822072 | G | A | 0.0251 | 0.0034 | 4.06E-12 |
| rs6450176 | G | A | 0.0254 | 0.0039 | 6.88E-10 |
| rs3936511 | A | G | 0.0308 | 0.0046 | 2.96E-09 |
| rs1936800 | C | T | 0.02 | 0.0034 | 3.06E-10 |
| rs3861397 | A | G | 0.024 | 0.0036 | 8.4E-11 |
| rs9457931 | A | G | 0.0552 | 0.0073 | 7.3E-13 |
| rs3823417 | A | G | 0.0285 | 0.0042 | 2.07E-11 |
| rs715299 | T | G | 0.024 | 0.0039 | 6.47E-09 |
| rs205262 | A | G | 0.0283 | 0.0039 | 3.88E-13 |
| rs998584 | C | A | 0.026 | 0.0038 | 2.27E-11 |
| rs11765979 | C | A | 0.0412 | 0.0048 | 3.11E-17 |
| rs13225097 | A | G | 0.0227 | 0.0039 | 4.33E-08 |
| rs17173637 | T | C | 0.0363 | 0.0057 | 1.9E-08 |
| rs4142995 | G | T | 0.0263 | 0.0037 | 9.37E-12 |
| rs4917014 | G | T | 0.0222 | 0.0036 | 1.03E-08 |
| rs702485 | G | A | 0.0243 | 0.0034 | 6.45E-12 |
| rs17145738 | T | C | 0.0408 | 0.0053 | 4.95E-13 |
| rs7014168 | G | A | 0.0267 | 0.0041 | 9.2E-10 |
| rs2293889 | G | T | 0.0312 | 0.0035 | 4.27E-17 |
| rs10808546 | T | C | 0.0409 | 0.0034 | 4.11E-30 |
| rs10087900 | G | A | 0.0231 | 0.0036 | 2.17E-09 |
| rs7016529 | T | C | 0.2186 | 0.0141 | 9.27E-45 |
| rs13702 | C | T | 0.1058 | 0.0038 | 1.3E-160 |
| rs13265868 | A | G | 0.0478 | 0.0035 | 6.1E-40 |
| rs16842 | T | C | 0.03 | 0.0038 | 3.82E-14 |
| rs4240624 | A | G | 0.0818 | 0.0058 | 1.32E-45 |
| rs2230808 | C | T | 0.0385 | 0.004 | 1.59E-20 |
| rs2853579 | T | G | 0.0499 | 0.0053 | 1.32E-19 |
| rs11789603 | T | C | 0.06 | 0.006 | 3.7E-21 |
| rs1883025 | C | T | 0.0698 | 0.0041 | 1.5E-65 |
| rs686030 | A | C | 0.055 | 0.0049 | 4.29E-27 |
| rs964184 | C | G | 0.1065 | 0.0071 | 6.09E-48 |
| rs6589581 | T | A | 0.0845 | 0.0137 | 2.26E-09 |

HDL, high-density lipoprotein; EA, effect allele; NEA, non-effect allele

**Supplementary Table S8. Genetic variants for triglycerides**

| **rsID** | **EA** | **NEA** | **Effect size** | **Standard error** | **P** |
| --- | --- | --- | --- | --- | --- |
| rs2250802 | A | G | 0.023 | 0.0037 | 1.21E-10 |
| rs1832007 | A | G | 0.0327 | 0.0047 | 1.72E-12 |
| rs10761762 | T | C | 0.027 | 0.0033 | 1.06E-17 |
| rs2068888 | G | A | 0.0241 | 0.0034 | 1.68E-11 |
| rs7350481 | T | C | 0.2254 | 0.0066 | 1E-200 |
| rs2187126 | A | G | 0.0543 | 0.0069 | 2.9E-15 |
| rs12294259 | T | C | 0.219 | 0.0069 | 1.8E-200 |
| rs9804646 | C | T | 0.0524 | 0.0064 | 2.82E-17 |
| rs5110 | A | C | 0.156 | 0.0124 | 2.14E-34 |
| rs7943309 | G | A | 0.0605 | 0.0087 | 1.16E-11 |
| rs10501321 | T | C | 0.0216 | 0.0035 | 1.41E-08 |
| rs174535 | C | T | 0.047 | 0.0034 | 1.73E-41 |
| rs11057408 | G | T | 0.0258 | 0.0035 | 2.05E-12 |
| rs1321257 | G | A | 0.0402 | 0.0034 | 5.99E-31 |
| rs11613352 | C | T | 0.028 | 0.0039 | 9.4E-14 |
| rs12748152 | T | C | 0.0372 | 0.0059 | 1.1E-09 |
| rs17513135 | T | C | 0.022 | 0.0039 | 1.63E-08 |
| rs16948098 | A | G | 0.08 | 0.0089 | 4.84E-17 |
| rs10468017 | T | C | 0.0379 | 0.0039 | 7.56E-21 |
| rs588136 | C | T | 0.0495 | 0.0041 | 3.37E-30 |
| rs3198697 | C | T | 0.0198 | 0.0034 | 2.21E-08 |
| rs4587594 | G | A | 0.0694 | 0.0035 | 3.5E-82 |
| rs749671 | G | A | 0.0211 | 0.0034 | 6.11E-10 |
| rs9930333 | G | T | 0.0208 | 0.0037 | 3.25E-08 |
| rs247616 | C | T | 0.0393 | 0.0037 | 1.12E-25 |
| rs5880 | C | G | 0.0475 | 0.0085 | 4.71E-08 |
| rs8077889 | C | A | 0.0252 | 0.0042 | 9.88E-09 |
| rs117877390 | C | T | 0.1099 | 0.0141 | 1.53E-09 |
| rs10401969 | T | C | 0.121 | 0.0065 | 9.7E-70 |
| rs731839 | G | A | 0.0224 | 0.0036 | 2.65E-09 |
| rs4803750 | G | A | 0.0423 | 0.007 | 9.52E-09 |
| rs7254892 | A | G | 0.1235 | 0.0106 | 1.4E-24 |
| rs439401 | C | T | 0.0659 | 0.0038 | 1.42E-66 |
| rs3760627 | C | T | 0.0189 | 0.0034 | 5.29E-09 |
| rs7248104 | G | A | 0.0222 | 0.0034 | 5.05E-10 |
| rs4804311 | A | G | 0.0392 | 0.006 | 1.49E-09 |
| rs6029143 | C | T | 0.0388 | 0.0071 | 4.93E-08 |
| rs4810479 | C | T | 0.0474 | 0.0038 | 2.07E-34 |
| rs6066141 | T | C | 0.0297 | 0.0053 | 2.34E-08 |
| rs13389219 | C | T | 0.0271 | 0.0034 | 2.6E-15 |
| rs676210 | G | A | 0.0733 | 0.0039 | 3.28E-71 |
| rs2972146 | T | G | 0.0281 | 0.0034 | 2.97E-15 |
| rs3761445 | A | G | 0.0232 | 0.0034 | 8.06E-12 |
| rs2304684 | T | C | 0.086 | 0.0127 | 5E-11 |
| rs1260326 | T | C | 0.1148 | 0.0034 | 1E-200 |
| rs11674085 | A | G | 0.0251 | 0.0044 | 2.86E-08 |
| rs10440120 | C | A | 0.0306 | 0.0044 | 5.34E-11 |
| rs645040 | T | G | 0.0293 | 0.004 | 1.83E-12 |
| rs6831256 | G | A | 0.0258 | 0.0035 | 1.6E-12 |
| rs442177 | T | G | 0.0309 | 0.0033 | 1.32E-18 |
| rs6882076 | C | T | 0.0286 | 0.0035 | 1.51E-15 |
| rs9686661 | T | C | 0.0379 | 0.0044 | 2.54E-16 |
| rs719726 | T | C | 0.0199 | 0.0035 | 2.49E-08 |
| rs634869 | T | C | 0.0272 | 0.0033 | 1.78E-14 |
| rs2665357 | C | A | 0.0212 | 0.0033 | 8.33E-10 |
| rs2508015 | G | A | 0.0252 | 0.0038 | 1.33E-10 |
| rs2247056 | C | T | 0.0378 | 0.0039 | 3.86E-21 |
| rs11752643 | T | C | 0.0802 | 0.0088 | 3.96E-19 |
| rs998584 | A | C | 0.0293 | 0.0037 | 3.42E-15 |
| rs38855 | A | G | 0.0187 | 0.0033 | 2.11E-08 |
| rs287621 | T | C | 0.0222 | 0.0037 | 7.67E-09 |
| rs4719841 | G | A | 0.0232 | 0.0034 | 8.86E-11 |
| rs11974409 | A | G | 0.0899 | 0.0042 | 1.4E-100 |
| rs72555385 | G | A | 0.0749 | 0.0124 | 3.76E-09 |
| rs6995541 | G | A | 0.0265 | 0.0037 | 1.34E-12 |
| rs1062219 | T | C | 0.0223 | 0.0034 | 1.69E-09 |
| rs2954022 | C | A | 0.078 | 0.0033 | 2.2E-113 |
| rs4871624 | G | T | 0.0254 | 0.0037 | 1.07E-11 |
| rs4921914 | C | T | 0.0353 | 0.004 | 4.87E-17 |
| rs7016529 | C | T | 0.1911 | 0.014 | 3.57E-35 |
| rs12678919 | A | G | 0.1702 | 0.0056 | 1.8E-199 |
| rs4738684 | A | G | 0.0205 | 0.0035 | 8.82E-09 |
| rs7005265 | T | A | 0.0336 | 0.0053 | 1.26E-10 |

EA, effect allele; NEA, non-effect allele

**Supplementary Table S9. One-sample MR analyses adjusted for all covariates**

| **Trait** | **MR effect size** | **MR 95% CI** | **MR p-value** |
| --- | --- | --- | --- |
| BMI | 1.15 | 1.03 to 1.27 | 0.011 |
| WC | 1.05 | 0.94 to 1.18 | 0.351 |
| SBP | 1.01 | 0.99 to 1.04 | 0.168 |
| Fasting glucose | 0.69 | 0.31 to 1.56 | 0.374 |
| HbA1c | 0.98 | 0.92 to 1.05 | 0.631 |
| LDL cholesterol | 1.55 | 1.19 to 2.03 | 0.001 |
| HDL cholesterol | 1.10 | 0.60 to 2.01 | 0.761 |
| Triglycerides | 1.19 | 0.92 to 1.54 | 0.187 |

Effect sizes represent odds ratio for every 1 standard deviation increment in exposure trait. The standard deviations are: 4.7 kg/m^2^, 13.4 cm, 20.5 mmHg, 1.2 mmol/L, 6.3 mmol/mol, 0.87 mmol/L, 0.38 mmol/L and 1.0 mmol/L, for BMI, WC, SBP, fasting glucose, HbA1c, LDL cholesterol, HDL cholesterol and triglycerides, respectively.

BMI, body mass index; WC, waist circumference; SBP, systolic blood pressure, HbA1c, glycated haemoglobin; LDL, low-density lipoprotein; HDL, high-density lipoprotein; CI, confidence interval; MR, Mendelian randomisation

**Supplementary Table S10. MR-Egger and Weight-median analyses**

| **Trait** | **MR-Egger effect size** | **MR-Egger 95% CI** | **MR-Egger p-value** | **Weighted-median effect size** | **Weighted-median 95% CI** | **Weighted-median p-value** |
| --- | --- | --- | --- | --- | --- | --- |
| BMI | 1.09 | 0.52 to 2.30 | 0.821 | 1.42 | 0.83 to 2.42 | 0.195 |
| WC | 1.03 | 0.27 to 3.84 | 0.969 | 1.28 | 0.77 to 2.11 | 0.339 |
| SBP | 0.72 | 0.27 to 1.90 | 0.502 | 0.95 | 0.49 to 1.82 | 0.872 |
| Fasting glucose | 1.12 | 0.54 to 2.30 | 0.761 | 0.84 | 0.44 to 1.63 | 0.613 |
| HbA1c | 0.001 | 0 to 2.07 | 0.081 | 0.04 | 0 to 42.33 | 0.364 |
| LDL cholesterol | 1.17 | 0.95 to 1.45 | 0.148 | 1.23 | 0.99 to 1.53 | 0.068 |
| HDL cholesterol | 1.23 | 0.91 to 1.64 | 0.178 | 1.10 | 0.85 to 1.44 | 0.467 |
| Triglycerides | 1.03 | 0.76 to 1.4 | 0.833 | 1.20 | 0.88 to 1.63 | 0.244 |

Effect sizes represent odds ratio for every 1 standard deviation increment in exposure trait.

BMI, body mass index; WC, waist circumference; SBP, systolic blood pressure, HbA1c, glycated haemoglobin; LDL, low-density lipoprotein; HDL, high-density lipoprotein; CI, confidence interval; MR, Mendelian randomisation

**Supplementary Table S11. One-sample MR analyses in a restricted sample (SARS-CoV-2 positive vs negative)**

| **Trait** | **MR effect size** | **MR 95% CI** | **MR p-value** |
| --- | --- | --- | --- |
| BMI | 1.05 | 0.96 to 1.14 | 0.293 |
| WC | 1.04 | 0.96 to 1.14 | 0.311 |
| SBP | 1.01 | 0.99 to 1.03 | 0.514 |
| Fasting glucose | 0.54 | 0.22 to 1.32 | 0.176 |
| HbA1c | 0.97 | 0.89 to 1.06 | 0.506 |
| LDL cholesterol | 1.74 | 1.22 to 2.47 | 0.002 |
| HDL cholesterol | 1.26 | 0.6 to 2.65 | 0.546 |
| Triglycerides | 1.26 | 0.94 to 1.69 | 0.127 |

Effect sizes represent odds ratio for every 1 standard deviation increment in exposure trait.

BMI, body mass index; WC, waist circumference; SBP, systolic blood pressure, HbA1c, glycated haemoglobin; LDL, low-density lipoprotein; HDL, high-density lipoprotein; CI, confidence interval; MR, Mendelian randomisation; SARS-CoV-2, severe acute respiratory syndrome coronavirus 2


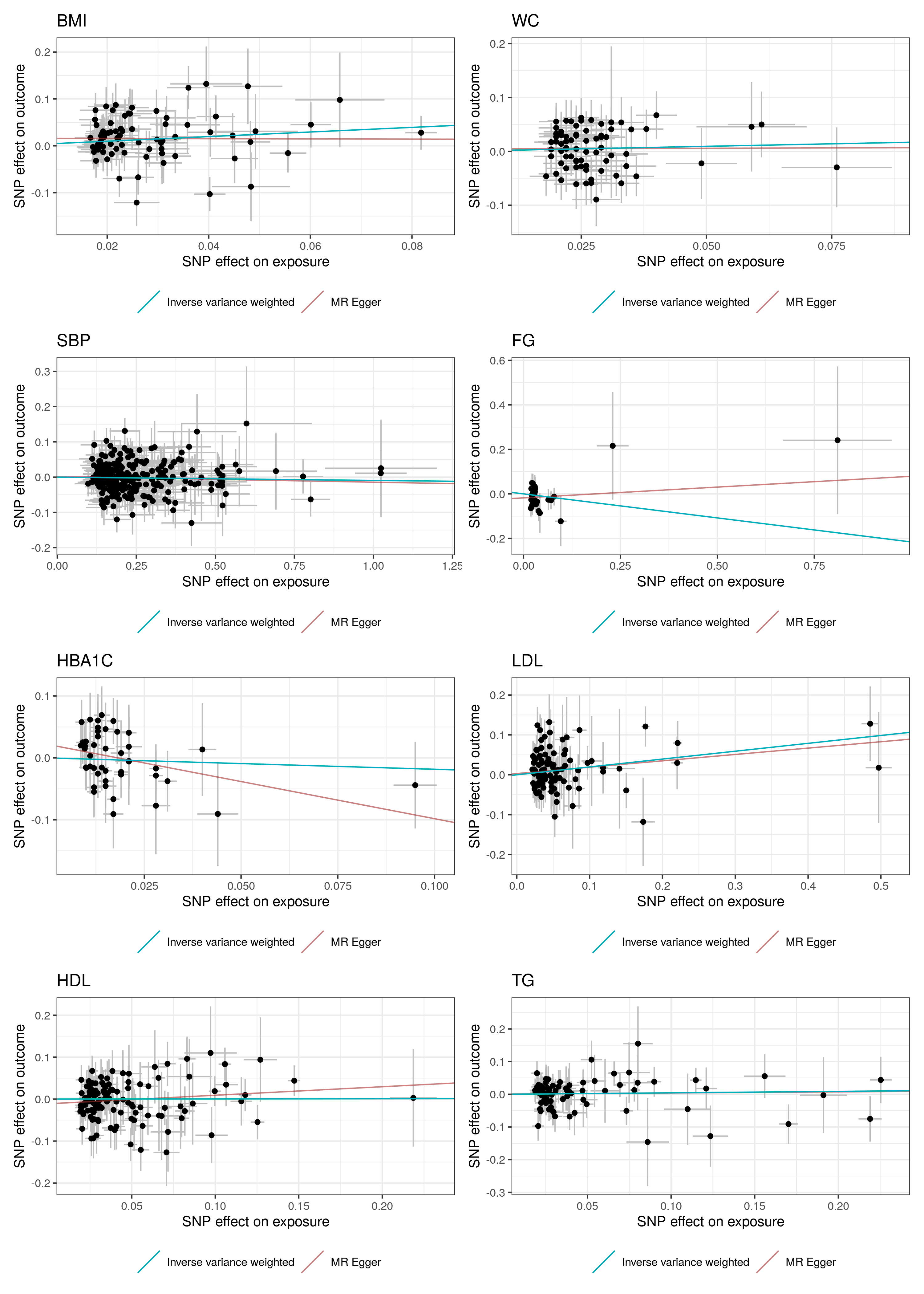


**Supplementary Figure S1. Scatter plots illustrating the associations between cardiometabolic traits and COVID-19 susceptibility**

BMI, body mass index; WC, waist circumference; SBP, systolic blood pressure, HbA1c, glycated haemoglobin; LDL, low-density lipoprotein; HDL, high-density lipoprotein; CI, confidence interval; MR, Mendelian randomisation

**References**

1. Tobin MD, Sheehan NA, Scurrah KJ, Burton PR. Adjusting for treatment effects in studies of quantitative traits: antihypertensive therapy and systolic blood pressure. *Stat Med*. 2005 Oct 15;**24**(19):2911–2935.

2. Friedewald WT, Levy RI, Fredrickson DS. Estimation of the concentration of low-density lipoprotein cholesterol in plasma, without use of the preparative ultracentrifuge. *Clinical chemistry*. 1972 Jun;**18**(6):499–502.

3. Jones PH, Davidson MH, Stein EA, et al. Comparison of the efficacy and safety of rosuvastatin versus atorvastatin, simvastatin, and pravastatin across doses (STELLAR* Trial). *The American journal of cardiology*. 2003 Jul 15;**92**(2):152–60.

4. Bycroft C, Freeman C, Petkova D, et al. The UK Biobank resource with deep phenotyping and genomic data. *Nature*. 2018 Oct;**562**(7726):203–209.

5. Locke AE, Kahali B, Berndt SI, et al. Genetic studies of body mass index yield new insights for obesity biology. *Nature*. Nature Publishing Group; 2015 Feb;**518**(7538):197–206.

6. Shungin D, Winkler TW, Croteau-Chonka DC, et al. New genetic loci link adipose and insulin biology to body fat distribution. *Nature*. Nature Publishing Group; 2015 Feb;**518**(7538):187–196.

7. Evangelou E, Warren HR, Mosen-Ansorena D, et al. Genetic analysis of over 1 million people identifies 535 new loci associated with blood pressure traits. *Nature Genetics*. Nature Publishing Group; 2018 Oct;**50**(10):1412–1425.

8. Manning AK, Hivert M-F, Scott RA, et al. A genome-wide approach accounting for body mass index identifies genetic variants influencing fasting glycemic traits and insulin resistance. *Nature Genetics*. Nature Publishing Group; 2012 Jun;**44**(6):659–669.

9. Wheeler E, Leong A, Liu C-T, et al. Impact of common genetic determinants of Hemoglobin A1c on type 2 diabetes risk and diagnosis in ancestrally diverse populations: A transethnic genome-wide meta-analysis. *PLOS Medicine*. Public Library of Science; 2017 Sep 12;**14**(9):e1002383.

10. Willer CJ, Schmidt EM, Sengupta S, et al. Discovery and refinement of loci associated with lipid levels. *Nature Genetics*. 2013 Nov;**45**(11):1274–1283.

11. The COVID-19 Host Genetics Initiative. The COVID-19 Host Genetics Initiative, a global initiative to elucidate the role of host genetic factors in susceptibility and severity of the SARS-CoV-2 virus pandemic. *European Journal of Human Genetics*. Nature Publishing Group; 2020 May 13;1–4.
